# Alkali Metal Triphenyl‐ and Trihydridosilanides Stabilized by a Macrocyclic Polyamine Ligand

**DOI:** 10.1002/chem.202000187

**Published:** 2020-02-18

**Authors:** Danny Schuhknecht, Valeri Leich, Thomas P. Spaniol, Iskander Douair, Laurent Maron, Jun Okuda

**Affiliations:** ^1^ Institute of Inorganic Chemistry RWTH Aachen University Landoltweg 1 52074 Aachen Germany; ^2^ CNRS INSA UPS, UMR 5215 LPCNO Université de Toulouse 135 avenue de Rangueil 31077 Toulouse France

**Keywords:** alkali metals, hydrogen storage, macrocyclic ligands, metal hydrides, silanides

## Abstract

Potassium silanide [KSiH_3_]_∞_ contains 4.2 wt % of hydrogen and has been intensely studied as hydrogen storage material. The macrocyclic ligand Me_4_TACD (1,4,7,10‐tetramethyl‐1,4,7,10‐tetraaminocyclododecane, **L**) stabilizes the full range of triphenylsilyl complexes [(**L**)MSiPh_3_]_*n*_ (M=Li–Cs), which react with H_2_ or PhSiH_3_ to form molecular [(**L**)MSiH_3_]_*n*_ that can be isolated in soluble form and fully characterized.

## Graphical abstract

**Silanides**: The macrocyclic polyamine ligand Me_4_TACD (**L**) stabilizes the full range of alkali metal triphenylsilanides [(**L**)MSiPh_3_]_*n*_ (M=Li–Cs) that have been studied in solution and in the solid state. They react with H_2_ or PhSiH_3_ to give molecular trihydridosilanides [(**L**)MSiH_3_] that are soluble in THF and can be regarded as model complexes for the hydrogen‐storage material [MSiH_3_]_∞_.

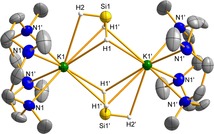

Metal hydrides are of current interest as hydrogen storage material, because H_2_ is considered as the only carbon‐free energy carrier.^1^ Suitable storage materials require defined thermodynamic properties of hydrogenation and dehydrogenation (enthalpy of hydrogenation −40 kJ mol^−1^), as well as reversibility of hydrogen storage under mild conditions (1–10 bar at temperatures of 40–100 °C).^2^ The alkali metal silanides [MSiH_3_]_∞_ contain high wt % of hydrogen (M=Li, 7.95; Na, 5.59; K, 4,31) and for the heavier K–Cs salts, reversible uptake and release of H_2_ has been shown.^3^ They can be synthesized by treating metal or metal alloys with hazardous SiH_4_ or PhSiH_3_,^4^ or more conveniently, by hydrogenolysis of the Zintl phase MSi.^3^, ^5^ The lighter [LiSiH_3_]_∞_ and [NaSiH_3_]_∞_ are less thermodynamically stable and not accessible from the Zintl phases that form stable MH and M_*x*_Si_*y*_ upon hydrogenolysis.3b, 6 Only few molecular complexes containing [SiH_3_]^−^ anions have been isolated from reactions involving SiH_4_ and studied in solution and in solid state.4b, 7 Alkali and alkaline earth metal silanides catalyze such reactions as hydrofunctionalization of olefins and pyridine,^8^ and are also active in the defluorination of organofluorides.^9^ Recently, we showed that the molecular complex [(Me_6_TREN)KSiPh_3_] reacts with H_2_ to give a soluble form of [α‐KSiH_3_]_∞_.^10^ Herein, we report the synthesis of the full range of alkali metal triphenylsilanides stabilized by the macrocyclic ligand Me_4_TACD (1,4,7,10‐tetramethyl‐1,4,7,10‐tetraaminocyclododecane, **L**) and their reactivity towards H_2_ and PhSiH_3_ to form molecular trihydridosilanes [(**L**)MSiH_3_]_*n*_.

Similar to previously reported crown ether adducts,8b Me_4_TACD stabilized alkali metal triphenylsilanides [(**L**)MSiPh_3_] (M=Li–Cs, **1**–**5**) were synthesized from the reaction of disilane Ph_3_SiSiMe_3_ with [MO*t*Bu] (M=Na–Cs) or in the case of lithium, with the more basic alkyl [LiCH_2_SiMe_3_] (Scheme 1). In all cases, the volatile by‐products (Me_3_Si)_2_CH_2_ or *t*BuOSiMe_3_ were readily removed under reduced pressure. Alternatively, ligand exchange from isolated THF adducts [(thf)_*x*_MSiPh_3_] (M=Li, K–Cs) with **L** also gave complexes **1** and **3**–**5**.

**Scheme 1:**
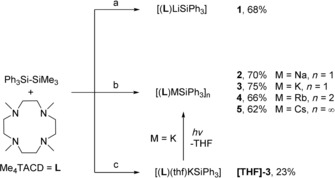
Synthesis of alkali metal triphenylsilanides [(**L**)MSiPh_3_]. a) 1. [LiCH_2_SiMe_3_], THF, 25 °C, (Me_3_Si)_2_CH_2_; 2. THF/*n*‐pentane, −30 °C. b) 1. [MO*t*Bu] (M=Na, K, Rb, Cs), THF, 25 °C, ‐*t*BuOSiMe_3_; 2. THF/*n*‐pentane, −30 °C. c) [KO*t*Bu], THF, 25 °C. 2. THF, 25 °C (low yield due to incomplete crystallization).

The triphenylsilanide complexes **1**–**5** were isolated in good yields from THF/*n*‐pentane at −30 °C. They are insoluble in aliphatic hydrocarbons, slightly soluble in benzene, but well soluble in THF; they were characterized by elemental analysis, NMR spectroscopy, and single‐crystal X‐ray diffraction. The solubility and stability of the light yellow compounds in THF decreased at room temperature from Li (*t*_1/2_>14 d) to Cs (*t*_1/2_≈12 h). The potassium complex also crystallized from neat THF as the solvate [(**L**)(thf)KSiPh_3_] (**[THF]‐3**) that gave amorphous [(**L**)KSiPh_3_] (**3**) under reduced pressure or extended storage at −30 °C, suggesting labile THF solvation. The ^1^H NMR spectra of compounds **1**–**5** in [D_8_]THF show a sharp singlet around *δ*=2.2 ppm for the ligand methyl groups and resonances for the [SiPh_3_]^−^ anion in the aromatic region with expected integral ratios. Although complexes **1** and **2** show two multiplets between *δ*=2.2 and 2.7 ppm for the methylene protons of the ligand, **3**–**5** containing the larger cations display a broad resonance at *δ*=2.4 ppm, indicating higher dynamics of the ligand ethylene bridges (Figure 1).^11^ Partial ligand dissociation due to larger ionic radii might also contribute to signal shift comparable to the free ligand.8e

**Figure 1:**
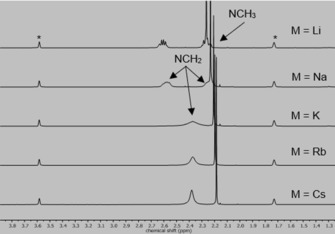
^1^H NMR spectra of complexes **1**–**5** in [D_8_]THF (*) at 25 °C.

Single crystals of the triphenylsilanides **1**–**5** were grown from THF/*n*‐pentane at −30 °C or in case of **[THF]‐3** from neat THF at room temperature. The smaller Li and Na complexes **1** and **2** crystallized in the acentric space group *Cc* with very similar lattice and structural parameters. Both cations show a distorted square pyramidal geometry (CN 5) with a κ^4^‐coordinated ligand, as well as a silicon‐bonded [SiPh_3_]^−^ anion (Figure 2 a). The M−Si distances of 2.796(6) Å (**1**) and 2.9501(10) Å (**2**) are well in the range of other light alkali metal silanides (Table 1).8b In both structures, one of the silanide phenyl groups is bent (Si‐C_i_‐C_o_‐C_m_≈170°, see the Supporting Information) due to Pauli repulsion, as was described for similar lithiosilanides.^12^ Potassium complex **[THF]‐3** shows a capped trigonal prismatic geometry (CN 6) with additional THF coordination and a significantly longer K−Si distance (3.4328(11) Å) due to higher CN and a larger ionic radius (see the Supporting Information). In contrast, the structure of unsolvated **3** (Figure 2 b) contains a non‐bonding K⋅⋅⋅Si interaction (3.9180(12) Å), but strong K‐η^6^‐arene coordination (average K−*C_Ph_*≈3.21 Å), as was previously observed in the complex [(Me_6_TREN)KSiPh_3_].^10^, ^13^ A switch from η^1^‐CH_2_ to η^6^‐aryl coordination depending on the cation has been commonly observed in alkali metal benzyl complexes.^14^ The heavier Rb and Cs complexes **4** and **5** show a more extended coordination sphere in the solid state: **4** crystallizes as dimer with bridging [SiPh_3_]^−^ units (Figure 2 c) similar to [(thf)MSiPh*t*Bu_2_]_2_ (M=Na, K),^15^ although **5** shows a one‐dimensional chain‐like structure (see the Supporting Information) with inverted triphenylsilanide anions coordinating only through Cs−C_Ph_ interactions (ØCs−C_Ph_≈3.66 Å).

**Figure 2:**
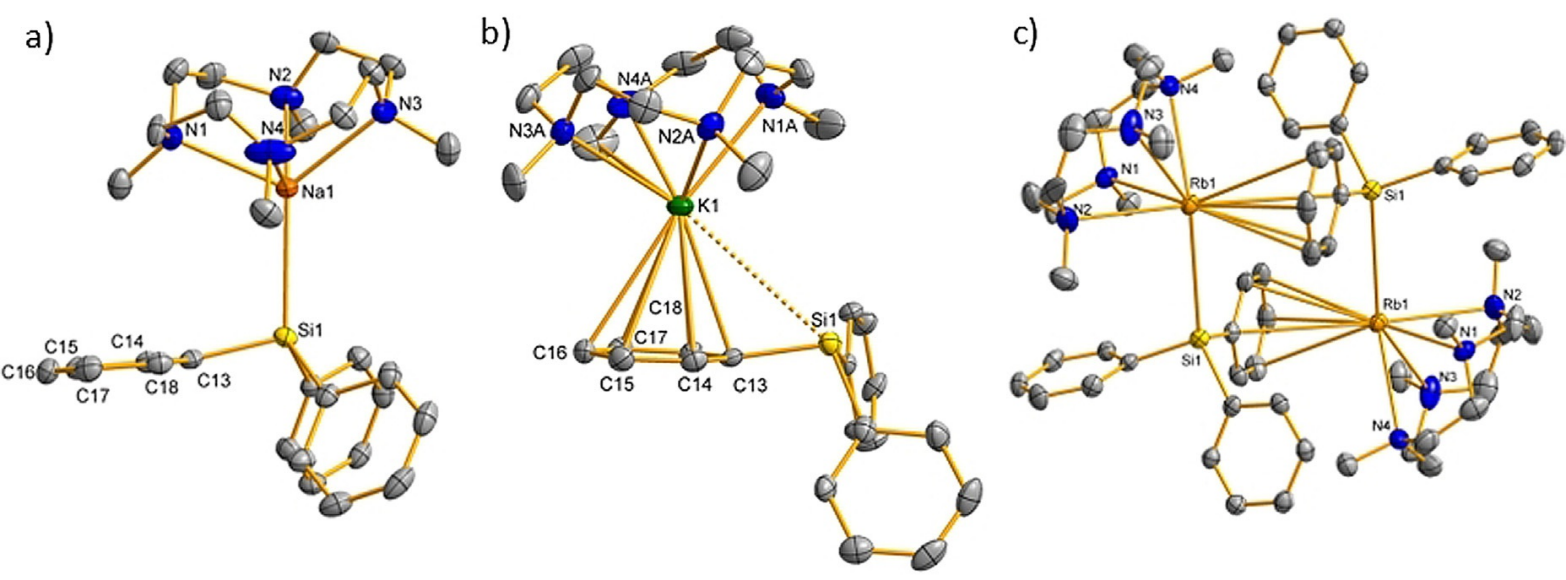
Molecular structures of a) [(**L**)NaSiPh_3_] (**2**), b) [(**L**)KSiPh_3_] (**3**) and c) [(**L**)RbSiPh_3_]_2_ (**4**). Displacement parameters are shown at a 50 % probability level. Hydrogen atoms are omitted for clarity.

**Table 1:** Selected structural parameters of the complexes **1**–**5** and **[THF]‐3**.

Complex (M)	Ionic radius^16^ [Å]/(CN)	ØN−M [Å]	M−Si [Å]	ØM−C_Ph_ [Å]	|Si‐C_i_‐C_o_‐C_m_| [°]
**1** (Li)	0.76/(5)	2.26	2.793(9)	–	169.4
**2** (Na)	1.00/(5)	2.48	2.9501(10)	–	169.6
**[THF]‐3** (K)	1.38/(6)	2.84	3.4328(11)	–	172.1
**3** (K)	1.46/(7)	2.80	(3.9180(12))	3.21	168.5
**4** (Rb)	1.61/(8)	3.04	3.775(2)	3.58	169.7
**5** (Cs)	1.74/(8)	3.16	(3.859(2))	3.66	173.8

Similar to the Me_6_TREN stabilized triphenylsilanide [(Me_6_TREN)KSiPh_3_],^10^ solutions of **1**–**5** in THF were pressurized with H_2_ (1 bar). Initial formation of the intermediate [SiHPh_2_]^−^ and [SiH_2_Ph]^−^, as well as benzene as by‐product, was observed after one and two days, respectively.^10^, ^17^ After seven days, the trihydridosilanide complexes [(**L**)MSiH_3_]_*n*_ **6**–**9** (M=Li–Rb) were formed. Cesium complex **5** also reacted with H_2_ in a stepwise fashion to form the trihydridosilanide and benzene, but free ligand **L** and precipitation of colorless [CsSiH_3_]_∞_ was observed after five days (Scheme 2).

**Scheme 2:**
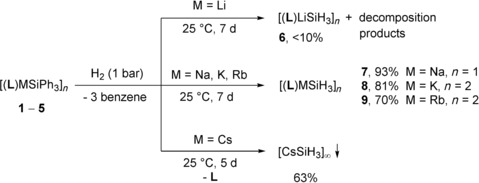
Synthesis of trihydridosilanides **6**–**9** by hydrogenolysis.

The selectivity of the hydrogenolysis of **1** was low and crystallization from THF/*n*‐pentane gave only low yield (≈10 %) of **6**. Other reaction products included neutral silanes H_*x*+1_SiPh_3−*x*_ (*x=*0–3) and decomposed ligand fragments, as was observed in the ^1^H NMR spectrum (see the Supporting Information). Highly reactive lithium bases have been shown to degrade amines in several ways.12b, 18 In contrast, the heavier complexes [(**L**)MSiH_3_]_*n*_ (M=Na, **7**; K, **8**; Rb, **9**) were formed selectively and isolated from THF/*n*‐pentane in good yield. Alternatively, the triphenylsilanides reacted with excess PhSiH_3_ to give complexes **6**–**9** after five minutes. The isolated yield for **6** was much higher (60 %), and no decomposition of the ligand was observed. ^1^H NMR spectroscopy revealed immediate scrambling of PhSiH_3_ to form Ph_2_SiH_2_ and SiH_4_ commonly observed catalyzed by strong bases (Scheme 3).11c, 19 Consecutive reaction of the in situ formed SiH_4_ and the triphenylsilanides **1**–**4** gave the [SiH_3_]^−^ complexes through silane–silanide exchange.^20^ Although complexes **7**–**9** are stable for weeks in solution and the solid state, complex **6** was significantly less stable (*t*_1/2_≈2 d).

**Scheme 3:**
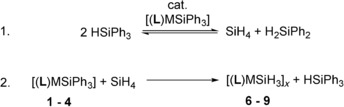
Reaction of complex **1**–**4** with PhSiH_3_.

Complexes **6**–**9** are insoluble in aliphatic and aromatic hydrocarbons, but slightly soluble in THF. They were analyzed by elemental analysis, NMR spectroscopy and for **7**–**9** by X‐ray diffraction. In [D_8_]THF, they showed signals for the methyl and methylene protons of the ligand with chemical shift, integral ratio and shape comparable to the respective triphenylsilanide complexes **1**–**4**. Each complex showed a sharp singlet in the area of *δ*=1.25–1.41 ppm with ^29^Si satellites (^1^ *J*_SiH_≈75 Hz) for the silanide anion [SiH_3_]^−^.4b, 21 A resonance between *δ*=−160 and −172 ppm in the ^29^Si{^1^H} NMR spectrum, as well as cross‐peaks in the ^1^H–^29^Si{^1^H} HSQC experiment further corroborated their formation.^22^ Unlike the more labile bounded Me_6_TREN, no dissociation of the macrocyclic Me_4_TACD ligand was observed during synthesis and isolation of **6**–**9**.^23^

Single crystals of **7**–**9** suitable for X‐ray analysis were grown from THF/benzene at 25 °C. The highly reactive sodium complex [(**L**)NaSiH_3_] (**7**) crystallized as monomer with square pyramidal coordination geometry similar to the parent compound **2** (see the Supporting Information).^7^ Several batches of crystals gave reproducibly low diffracting crystals, which prevented locating the hydrides in a Fourier difference map and assignment of the κSi or κ^3^H coordination mode due to similar expected M−Si distances.^7^ The heavier silanides [(**L**)M(μ‐SiH_3_)]_2_ (M=K (**8**); Rb (**9**)) crystallized as dimers in the tetragonal space group *P*4_2_/*mnm* with nearly identical lattice and structural parameters. The interatomic K1⋅⋅⋅K1 and K1⋅⋅⋅Si1 distances in **8** are comparable to those in the β‐KSiH_3_ phase.^24^ The hydrides were located in a Fourier difference map and refined. The Si–H and K–H interactions are comparable to those in the crown ether adduct,^24^, ^25^ but should be treated with care due to the heavy metals (Figure 3).

**Figure 3:**
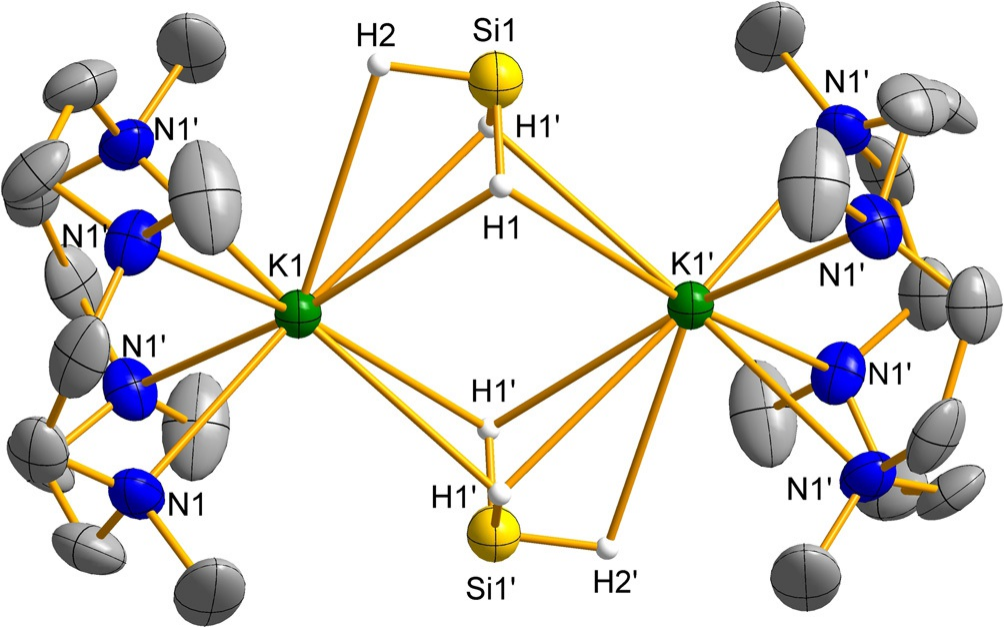
Molecular structure of [(**L**)KSiH_3_]_2_ (**8**). Displacement parameters are shown at a 50 % probability level. Hydrogen atoms and solvent in the lattice are omitted for clarity. Selected interatomic distances [Å]: K1−N1 2.862(2), K1⋅⋅⋅K1’ 4.4691(17), K1⋅⋅⋅Si1 3.5625(13), K1−H1 3.06(2), K1−H2 3.12(7), Si1−H1 1.31(3), Si1−H2 1.32(7).

The structures of **7** and **8** were further investigated by DFT calculations (B3PW91). In both cases, the optimized structures compare well with the ones observed in the solid state (see the Supporting Information). Two possible structures of **7** were optimized with a low energy difference of approximately 2 kcal mol^−1^ in favor of the κSi‐bonded [SiH_3_]^−^ group. For both structures, natural bonding analysis (NBO) was carried out. For the most stable complex, the Na−Si Wiberg bond index (WBI) is 0.45 indicating a polar covalent Na−Si bond. The second isomer contains an inverted hydride‐bridged [κ^3^ *H*‐SiH_3_]^−^ ligand with a significantly lower Na−Si WBI (0.12), in line with a weaker Na–Si interaction. The stability of the latter is therefore explained by the second‐order donor–acceptor interaction, in which strong donations of approximately 43 kcal mol^−1^ from the covalent Si−H bonds (WBI 0.86) to the sodium cation are found. As was observed in the solid state for [α‐MSiH_3_]_∞_, both structures might be present and interchangeable, preventing location of the hydrides in the crystal structure.4b, 24 In contrast, the dimeric potassium complex **8** is significantly more stable with bridging [κ^3^ *H*‐SiH_3_] units (23.3 kcal mol^−1^). For the dimer, the Si‐K WBI is expectedly low (0.06). For the inverted hydride‐bridged isomer of **7**, donations from the covalent Si−H bonds (WBI 0.89) towards the cations (about 22 kcal mol^−1^ each) ensure the stabilization of the complex.

In conclusion, the macrocyclic ligand Me_4_TACD stabilizes the full range of alkali metal triphenylsilanides that show the structural trend from Si coordination in **1** and **2** towards M⋅⋅⋅*C_Ph_* interactions in the heavier analogues **4** and **5**. Both coordination modes were observed in the potassium complexes **3** and **[THF]‐3** depending on the crystallization conditions. The [(**L**)MSiPh_3_] complexes reacted with H_2_ in THF at ambient conditions to give trihydridosilanides [(**L**)MSiH_3_]_*x*_ and benzene. Both Na and K complexes showed an ionic structure with Si−H donation towards the metal center. In the case of Li, strong basicity hindered the selective formation by hydrogenolysis, however, silane–silanide exchange from in situ formed SiH_4_ gave the lightest silanide [(**L**)LiSiH_3_] in moderate yield. As was observed for early main‐group metal hydrides,^26^ smaller aggregates could show improved dehydrogenation kinetics, which will be further investigated.

## Conflict of interest

The authors declare no conflict of interest.

## Supporting information

As a service to our authors and readers, this journal provides supporting information supplied by the authors. Such materials are peer reviewed and may be re‐organized for online delivery, but are not copy‐edited or typeset. Technical support issues arising from supporting information (other than missing files) should be addressed to the authors.

Supplementary
