# Supplementary material for: Alkali Metal Triphenyl‐ and Trihydridosilanides Stabilized by a Macrocyclic Polyamine Ligand
Source: Chemistry. 2020 Feb 18;26(13):2821–5. doi: 10.1002/chem.202000187 (PMC7079104; doi:10.1002/chem.202000187)
Supplement: Supplementary file 1 — Supplementary [file CHEM-26-2821-s001.pdf]

CHEMISTRY
A European Journal

Supporting Information

Alkali Metal Triphenyl- and Trihydridosilanides Stabilized by a Macrocyclic Polyamine Ligand

Danny Schuhknecht,[a] Valeri Leich,[a] Thomas P. Spaniol,[a] Iskander Douair,[b] Laurent Maron,[b] and Jun Okuda*[a]

chem_202000187_sm_miscellaneous_information.pdf

Table of Contents

1. General Remarks S2

2. Synthetic Procedures S3

3. X-Ray Crystallography.. S37

4. DFT Calculations.. S43

5. References.. S47

1. General Remarks

All operations were performed under an inert atmosphere of dry argon using standard Schlenk line or glovebox techniques. [D_8]THF was distilled under argon from sodium/benzophenone ketyl prior to use. THF and *n*-pentane were purified using a MB SPS-800 solvent purification system. Elemental analyses were performed on an *elementar vario EL* machine. In several instances, the results were not satisfactory, possibly due to incomplete combustion and high air- and moisture sensitivity.[S1] ^{1}H, ^{13}C{^{1}H}, ^{7}Li{^{1}H}, ^{23}Na{^{1}H} and ^{29}Si{^{1}H} NMR spectra were recorded on a *Bruker Avance II 400* or a *Bruker Avance III HD 400* spectrometer at 25 °C in J. Young type NMR tubes. Chemical shifts for ^{1}H and ^{13}C{^{1}H} spectra were referenced internally using the residual solvent resonance and are reported relative to TMS. ^{7}Li{^{1}H}, ^{23}Na{^{1}H} and ^{29}Si{^{1}H} NMR spectra were referenced externally to LiCl in D_2O, NaCl in D_2O or TMS in [D_8]THF, respectively. The resonances in the ^{1}H and ^{13}C{^{1}H} NMR spectra were assigned on the basis of two-dimensional NMR experiments (COSY, HSQC, HMBC). All reactions were carried out using glass coated stir bars. 1,4,7,10-tetramethyl-1,4,7,10-tetraazacyclodocecane (Me_4TACD),[S2] $Ph_3SiSiMe_3$,[S3] [$LiCH_2SiMe_3$][S4] [RbO^{t}Bu][S5] and [CsO^{t}Bu][S5] were prepared according to literature procedures. [NaO^{t}Bu] and [KO^{t}Bu] (sublimed grade) were commercially available and sublimed before use.

2. Synthetic Procedures

2.1. Synthesis of [(Me_4TACD)LiSiPh_3] (1)

$Ph_3SiSiMe_3$ (332 mg, 1.00 mmol) and [$LiCH_2SiMe_3$] (94 mg, 1.0 mmol) were dissolved in THF (4 mL) and stirred at 25 °C. After 30 min, Me_4TACD (230 mg, 1.00 mmol) was added dropwise. After 16 h the solution was reduced *in vacuo* to 2 mL, layered with *n*-pentane (2 mL) and stored at -30 °C. Single crystals grew within 16 h. The supernatant was decanted of, the yellow solid was washed with *n*-pentane (3 x 3 mL) and dried *in vacuo* to give [(Me_4TACD)LiSiPh_3] (**1**) (336 mg, 0.68 mmol, 68%) as yellow microcrystals.

Single crystals of [(Me_4TACD)LiSiPh_3] (**1**) were grown from a THF/*n*-pentane solution at -30 °C.

^{1}H NMR (400 MHz, [D_8]THF, 25 °C): δ = 2.20 – 2.35 (m, 20 H, NCH_2 + NCH_3), 2.55 – 2.67 (m, 8 H, NCH_2), 6.81 – 6.88 (m, 3 H, *para*-Ph), 6.93 – 7.00 (m, 6 H, *meta*-Ph), 7.39 – 7.45 (m, 6 H, *ortho*-Ph) ppm.

^{13}C{^{1}H} NMR (101 MHz, [D_8]THF, 25 °C): δ = 45.51 (CH_3), 54.85 (CH_2), 124.23 (*para*-Ph), 126.77 (*meta*-Ph), 137.11 (*ortho*-Ph), 157.40 (*ipso*-Ph) ppm.

^{29}Si{^{1}H} NMR (80 MHz, [D_8]THF, 25 °C): δ = –10.32 ppm.

^{7}Li{^{1}H} NMR (155.5 MHz, [D_8]THF, 25 °C): δ = 0.97 ppm.

Anal. Calcd for $C_{30}H_{43}N_4LiSi$ (494.73 g·mol^{-1}): C, 72.83; H, 8.76; N, 11.33. Found: C, 71.50; H, 8.67; N, 11.30%.

2.2. NMR spectra of [(Me_4TACD)LiSiPh$_3$] (1)

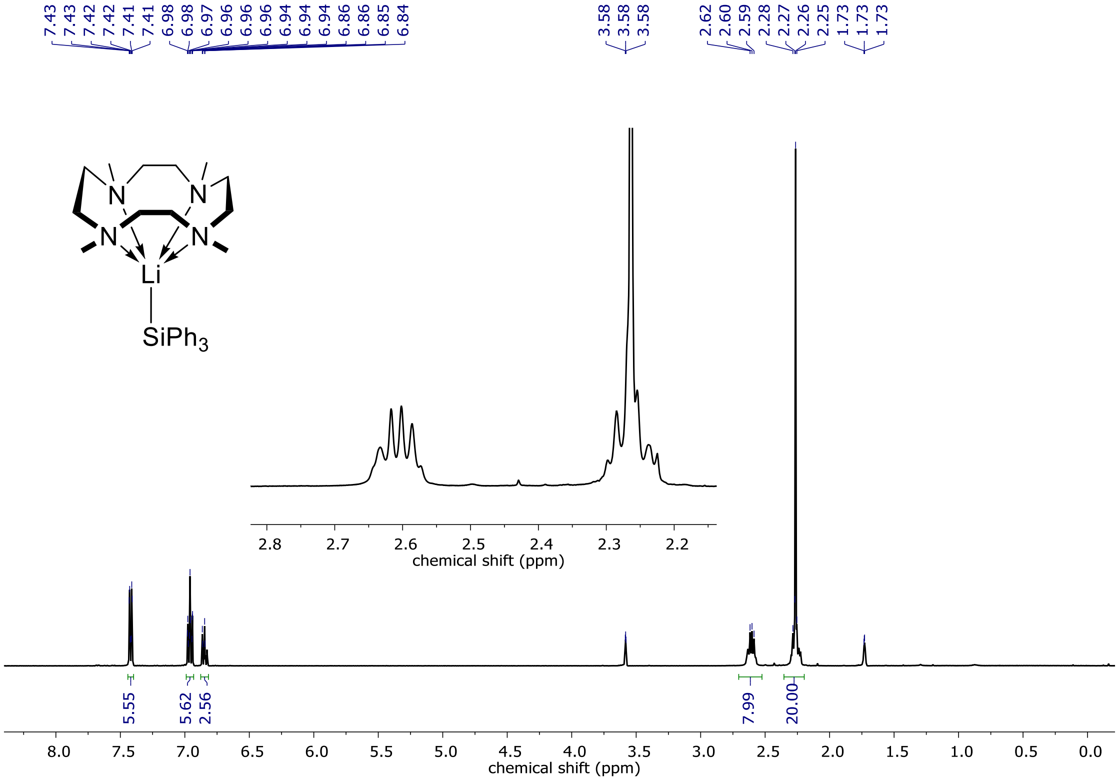

Figure S1. ^{1}H NMR (400 MHz, [D$_8$]THF, 25 °C) of [(Me_4TACD)LiSiPh$_3$] (**1**).

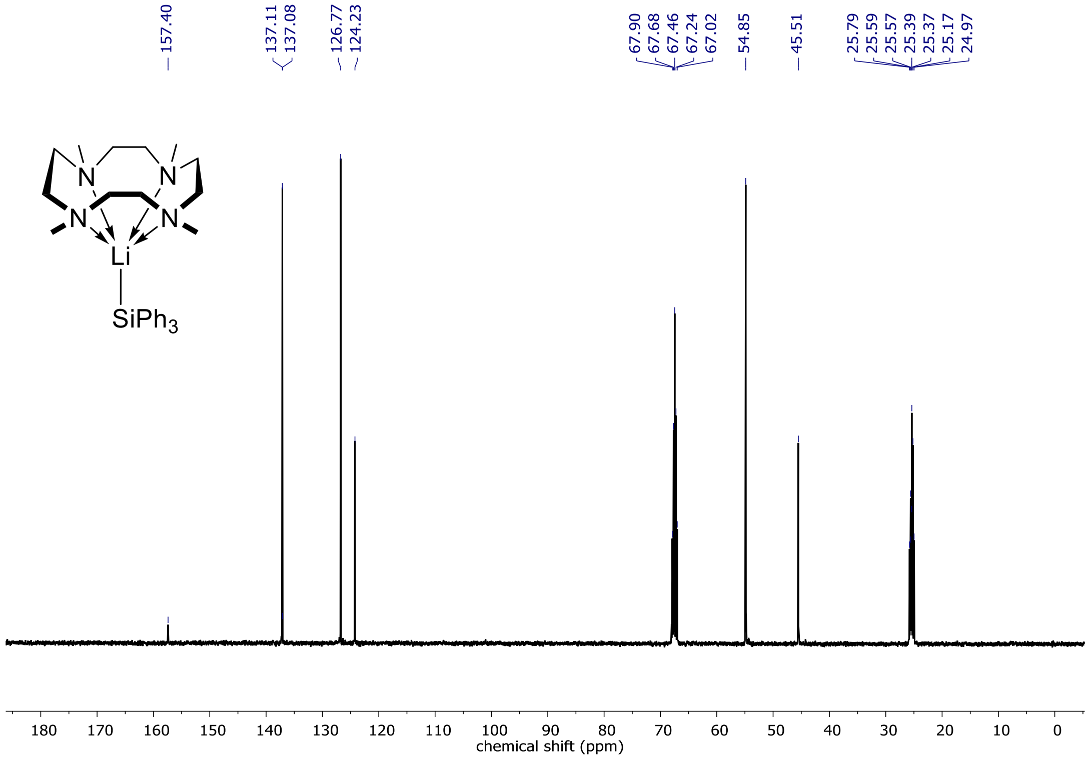

Figure S2. ^{13}C{^{1}H} NMR (101 MHz, [D$_8$]THF, 25 °C) of [(Me_4TACD)LiSiPh$_3$] (**1**).

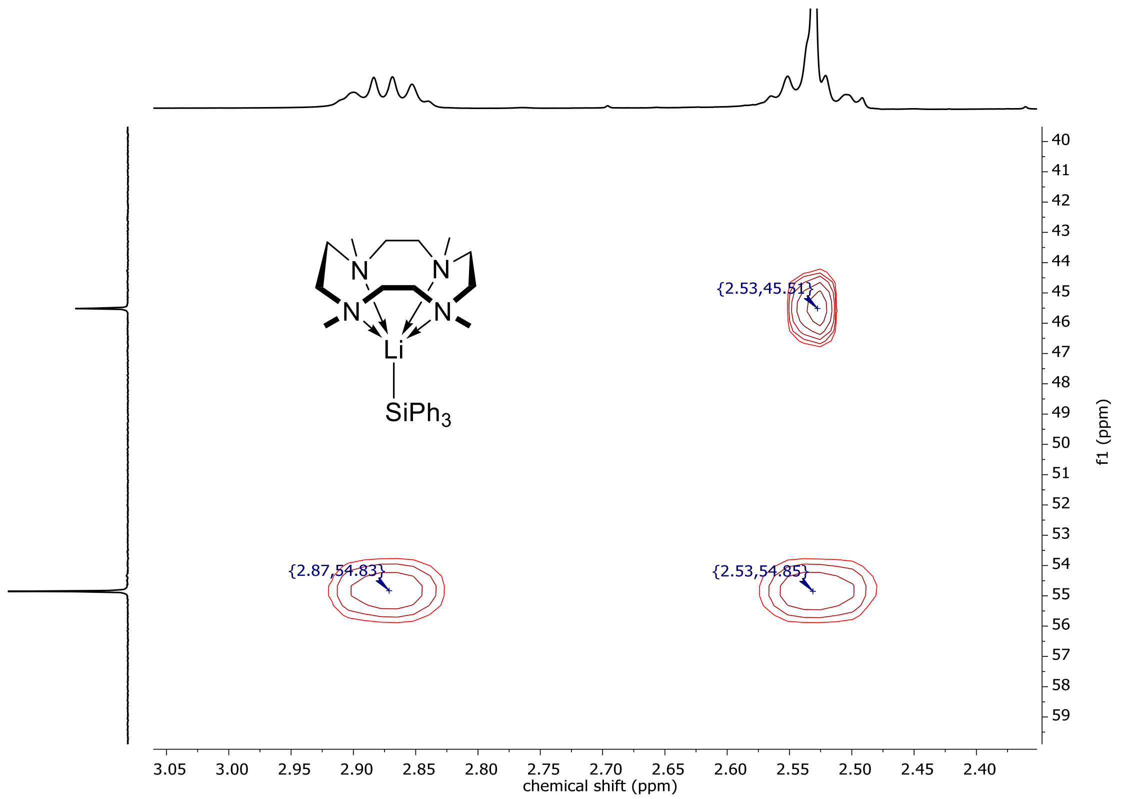

Figure S3. ^{1}H-$^{13}C\{^{1}H\}$ HSQC NMR (400/101 MHz, [D$_8$]THF, 25 °C) of [(Me$_4$TACD)LiSiPh$_3$] (**1**).

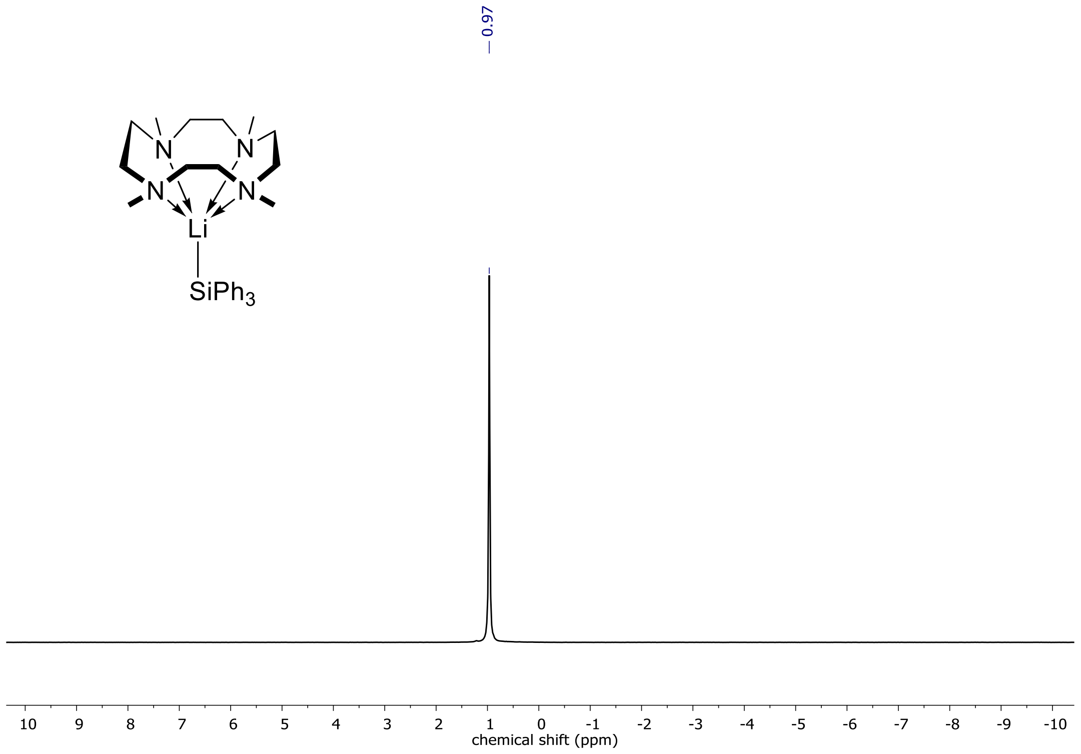

Figure S4. $^{7}Li\{^{1}H\}$ NMR (155.5 MHz, [D$_8$]THF, 25 °C) of [(Me$_4$TACD)LiSiPh$_3$] (**1**).

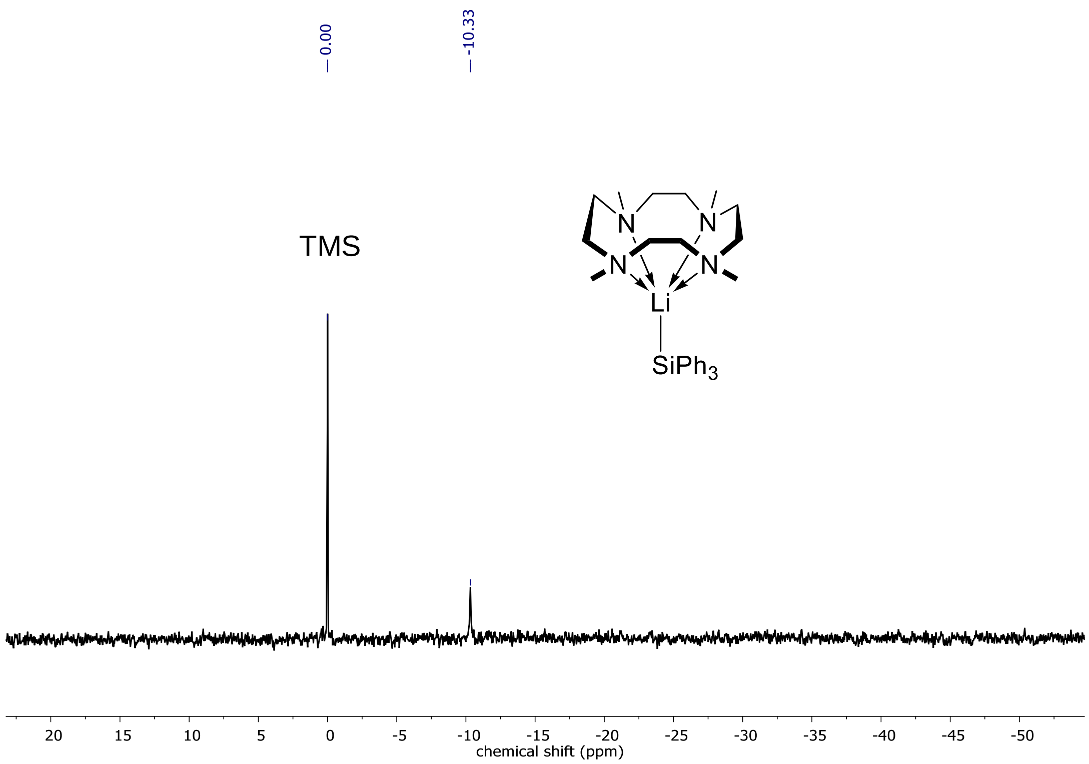

Figure S5. $^{29}Si\{^{1}H\}$ NMR (80 MHz, $[D_8]THF$, 25 °C) of $[(Me_4TACD)LiSiPh_3]$ (**1**).

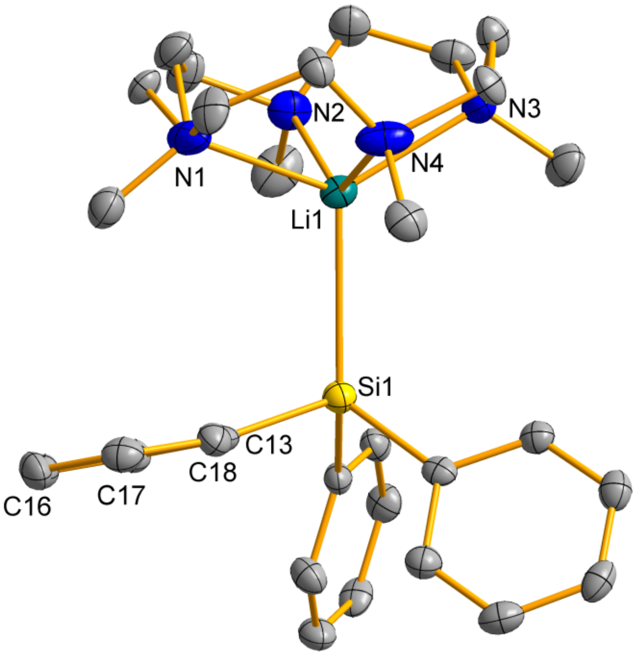

Figure S6. Molecular structures of $[(Me_4TACD)LiSiPh_3]$ (**1**). Displacement parameters are shown at a 50% probability level. Hydrogen atoms are omitted for clarity. Selected interatomic distances [Å] and angles [°]: Li1-Si1 2.796(6), Li1-N1 2.277(7), Li1-N2 2.219(6), Li1-N3 2.298(6), Li1-N4 2.231(6), Si1-C13-C18-C17 169.3(3).

2.3. Synthesis of [(Me$_4$TACD)NaSiPh$_3$] (2)

Ph$_3$SiSiMe$_3$ (666 mg, 2.0 mmol) and NaOtBu (192 mg, 2.0 mmol) were dissolved in THF (8 mL) and stirred at 25 °C. After 30 min, Me$_4$TACD (460 mg, 2.0 mmol) was added dropwise. After 16 h the solution was reduced *in vacuo* to ca 4 mL, layered with *n*-pentane (4 mL) and stored at –30 °C. After 16 h, yellow microcrystals formed. The supernatant was decanted off, the solid washed with n-pentane (3 x 5 mL) and dried *in vacuo* to give [(Me$_4$TACD)NaSiPh$_3$] (**2**) (707 mg, 1.4 mmol, 70%) as yellow microcrystals.

Single crystals of [(Me$_4$TACD)NaSiPh$_3$] (**2**) were grown from a THF/*n*-pentane solution at -30 °C.

^{1}H NMR (400 MHz, [D$_8$]THF, 25 °C): δ = 2.30 – 2.18 (m, 20 H, NCH_2 + NCH_3), 2.64 – 2.51 (m, 8 H, NCH_2), 6.93 – 6.83 (m, 3 H, *para*-Ph), 7.02 – 6.95 (m, 6 H, *meta*-Ph), 7.42 – 7.34 (m, 6 H, *ortho*-Ph) ppm.

^{13}C{^{1}H} NMR (101 MHz, [D$_8$]THF, 25 °C): δ = 44.26 (CH$_3$), 54.36 (CH$_2$), 124.50 (*para*-Ph), 126.90 (*meta*-Ph), 137.17 (*ortho*-Ph), 159.79 (*ipso*-Ph) ppm.

^{23}Na{^{1}H} NMR (106 MHz, [D$_8$]THF, 25 °C): δ = 27 ($w_{1/2}$ = 600 Hz) ppm .

^{29}Si{^{1}H} NMR (80 MHz, [D$_8$]THF, 25 °C): δ = –4.62 ppm.

Anal. Calcd for $C_{30}H_{43}N_4NaSi$ (510.78 g·mol^{-1}): C, 70.55; H, 8.49; N, 10.97. Found: C, 69.72; H, 8.72; N, 10.81%.

2.4. NMR spectra of [(Me_4TACD)NaSiPh_3] (2)

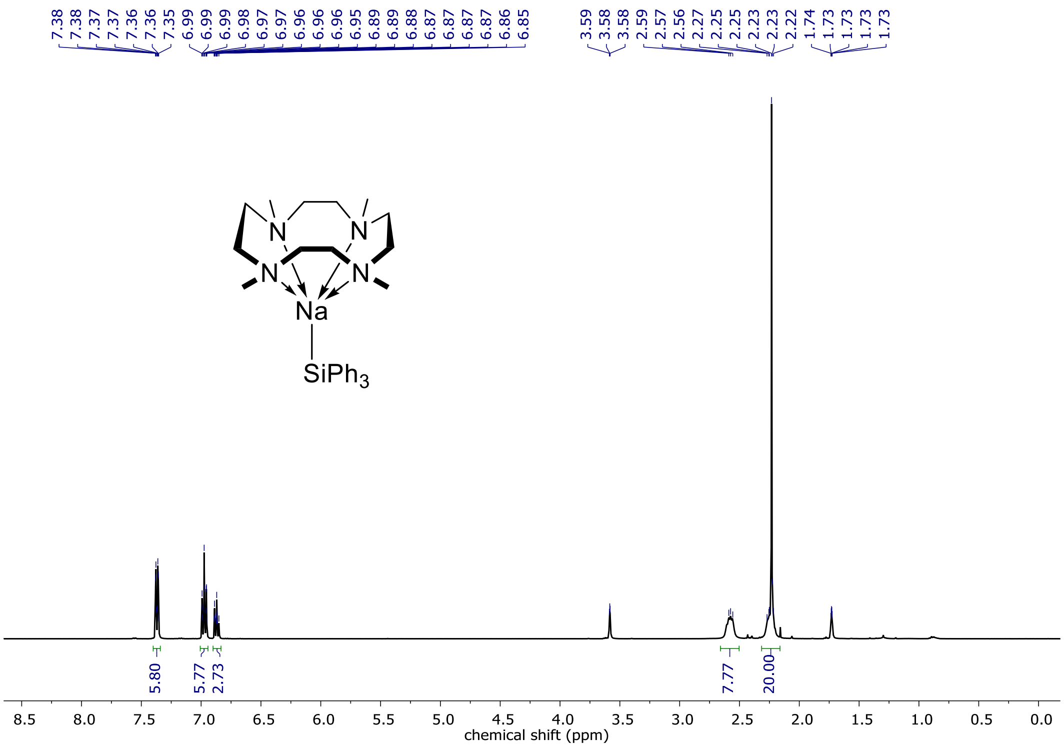

Figure S7. ^{1}H NMR (400 MHz, [D_8]THF, 25 °C) of [(Me_4TACD)NaSiPh_3] (**2**).

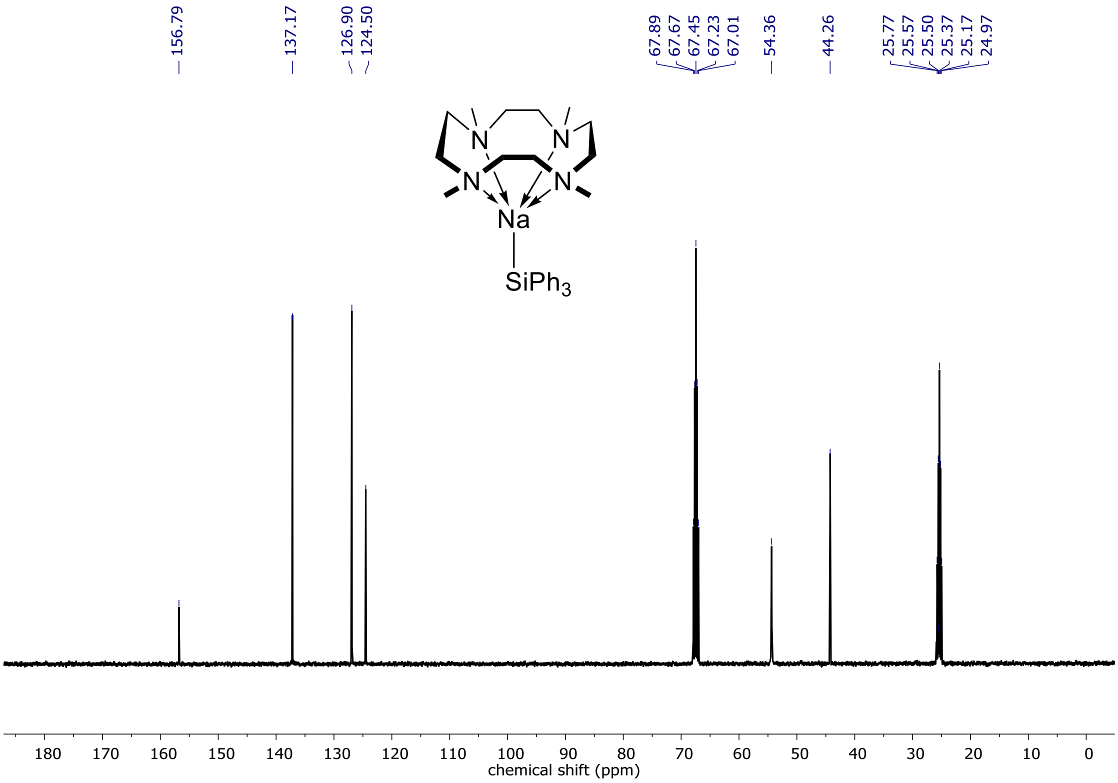

Figure S8. ^{13}C{^{1}H} NMR (101 MHz, [D_8]THF, 25 °C) of [(Me_4TACD)NaSiPh_3] (**2**).

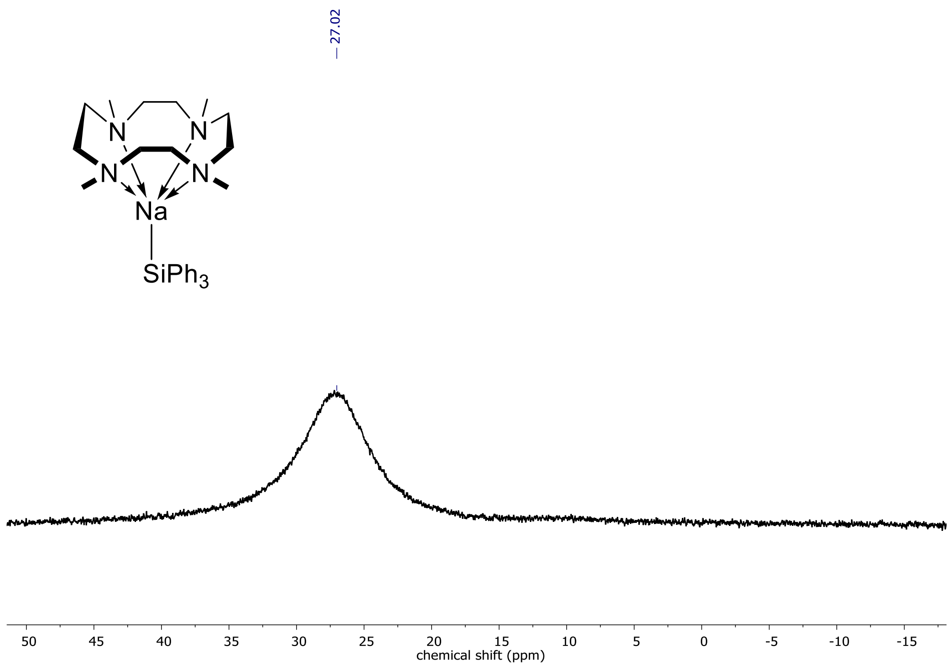

Figure S9. $^{23}Na\{^{1}H\}$ NMR (106 MHz, $[D_8]$THF, 25 °C) of $[(Me_4TACD)NaSiPh_3]$ (**2**).

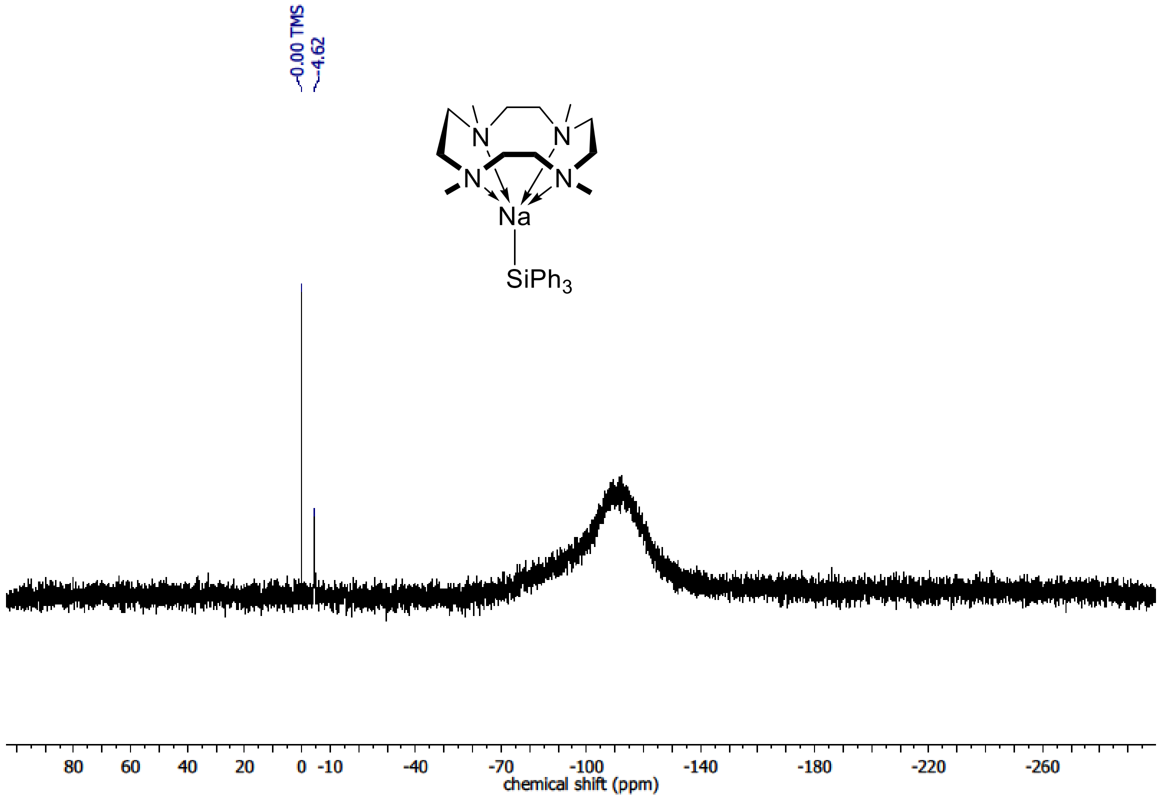

Figure S10. $^{29}Si\{^{1}H\}$ NMR (80 MHz, $[D_8]$THF, 25 °C) of $[(Me_4TACD)NaSiPh_3]$ (**2**).

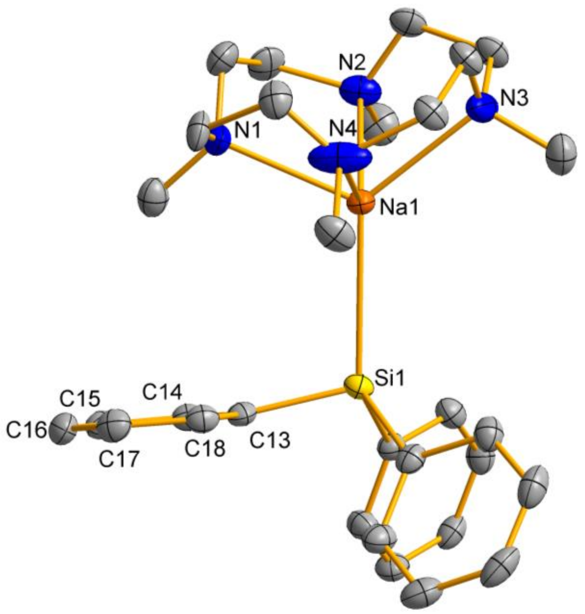

Figure S11. Molecular structures of [(Me_4TACD)NaSiPh_3] (**2**). Displacement parameters are shown at a 50% probability level. Hydrogen atoms are omitted for clarity. Selected interatomic distances [Å] and angles [°]: Na1-Si1 2.9501(10), Na1-N1 2.480(2), Na1-N2 2.472(2), Na1-N3 2.492(2), Na1-N4 2.474(2), Si1-C13-C18-C17 169.57(17).

2.5. Synthesis of [(Me$_4$TACD)KSiPh$_3$] (3) and [(Me$_4$TACD)K(thf)SiPh$_3$] [THF]-3

Method A: Ph$_3$SiSiMe$_3$ (332 mg, 1.0 mmol) and KOtBu (112 mg, 1.0 mmol) were dissolved in THF (5 mL) in stirred at 25 °C. After 30 min, Me$_4$TACD (230 mg, 1.0 mmol) was added dropwise. After 4 h the solution was reduced *in vacuo* to ca 3 mL, layered with *n*-pentane (4 mL) and stored at –30 °C. After 16 h, orange microcrystals formed. The supernatant was decanted off, the solid washed with n-pentane (3 x 2 mL) and dried *in vacuo* to give [(Me$_4$TACD)KSiPh$_3$] (**2**) (394 mg, 0.75 mmol, 75%) as orange microcrystals. Alternative workup: Crystallization from neat THF gave [(Me$_4$TACD)(thf)$_{0,75}$KSiPh$_3$] (**[THF]-3**) (130 mg, 0.23 mmol, 23 %) after work-up at 25 °C. Low yield is due to incomplete crystallization.

Method B: Me$_4$TACD (228 mg, 1.0 mmol) was added to a solution of [(thf)KSiPh$_3$] (371 mg, 1.0 mmol) in THF (5 mL) and stirred at 25 °C. After 5 min, the solution was reduced *in vacuo* to ca 3 mL, layered with *n*-pentane (4 mL) and stored at –30 °C. After 16 h, orange microcrystals formed. The supernatant was decanted off, the solid washed with *n*-pentane (3×3 mL) and dried *in vacuo* to give [(Me$_4$TACD)KSiPh$_3$] (**3**) (450 mg, 0.85 mmol, 85%) as orange crystals. Single crystals of [(Me$_4$TACD)KSiPh$_3$] (**3**) were grown from a THF/*n*-pentane solution at -30 °C.

^{1}H NMR (400 MHz, [D$_8$]THF, 25 °C): δ = 2.21 (s, 12 H, NCH_3), 2.22 – 2.70 (br, 16 H, NCH_2), 6.85 – 6.79 (m, 3 H, *para*-Ph), 6.96 – 6.91 (m, 6 H, *meta*-Ph), 7.36 – 7.32 (m, 6 H, *ortho*-Ph) ppm.

^{13}C{^{1}H} NMR (101 MHz, [D$_8$]THF, 25 °C): δ = 44.46 (CH$_3$), 54.93 (CH$_2$), 123.85 (*para*-Ph), 126.81 (*meta*-Ph), 137.01 (*ortho*-Ph), 158.88(*ipso*-Ph) ppm.

^{29}Si{^{1}H} NMR (80 MHz, [D$_8$]THF, 25 °C): δ = –7.24 ppm.

Anal. Calcd for $C_{30}H_{43}N_4KSi$ (526.89 g·mol^{-1}): C, 68.39; H, 8.23; N, 10.63. Found: C, 67.71; H, 8.07; N, 11.02.

Single crystals of [(Me$_4$TACD)(thf)KSiPh$_3$] (**[THF]-3**) were grown from a concentrated THF solution at 25 °C.

^{1}H NMR (400 MHz, [D$_8$]THF, 25 °C): δ = 1.75 – 1.80 (m, 4 H, THF), 2.21 (s, 12 H, NCH_3), 2.22 – 2.70 (br, 16 H, NCH_2), 3.60 – 3.65 (m, 4 H, THF), 6.79 – 6.85 (m, 3 H, *para*-Ph), 6.91 – 6.97 (m, 6 H, *meta*-Ph), 7.32 – 7.37 (m, 6 H, *ortho*-Ph) ppm.

Anal. Calcd for $C_{34}H_{51}N_4KOSi$ (598.99 g·mol^{-1}): C, 68.18; H, 8.58; N, 9.35. Found: C, 66.89; H, 8.15; N, 9.91.

2.6. NMR spectra of [(Me$_4$TACD)SiPh$_3$] (3) and [(Me$_4$TACD)(thf)KSiPh$_3$] ([THF]-3)

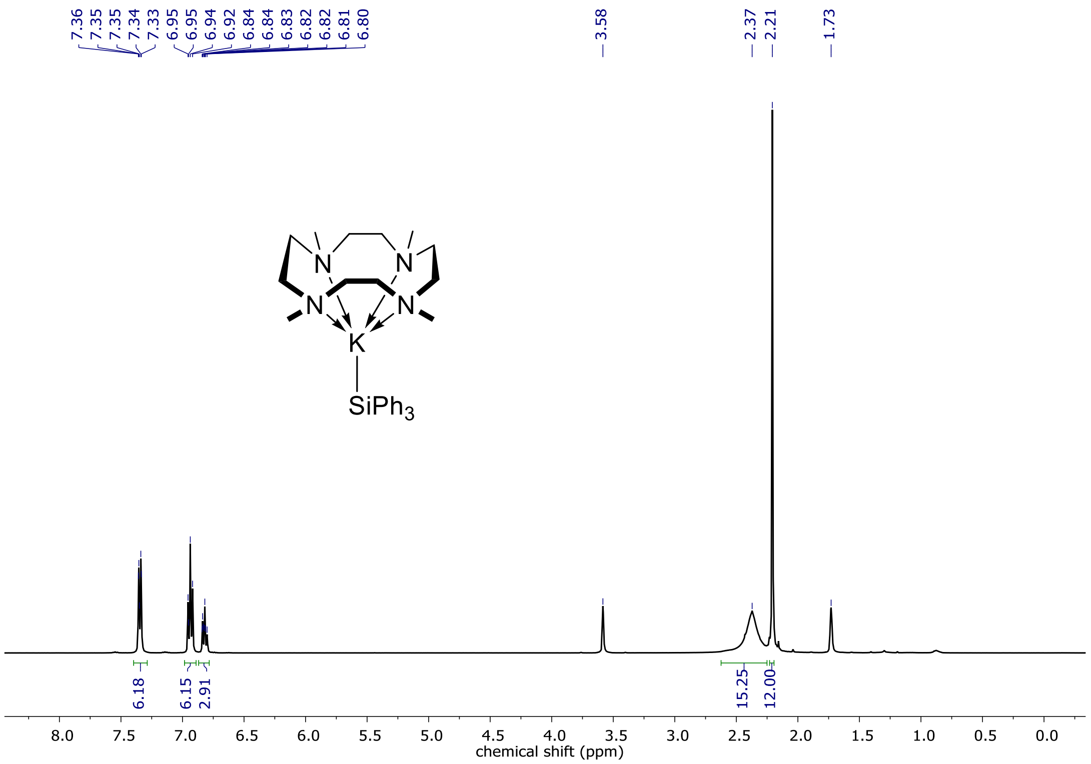

Figure S12. ^{1}H NMR (400 MHz, [D$_8$]THF, 25 °C) of [K(Me$_4$TACD)SiPh$_3$] (**3**).

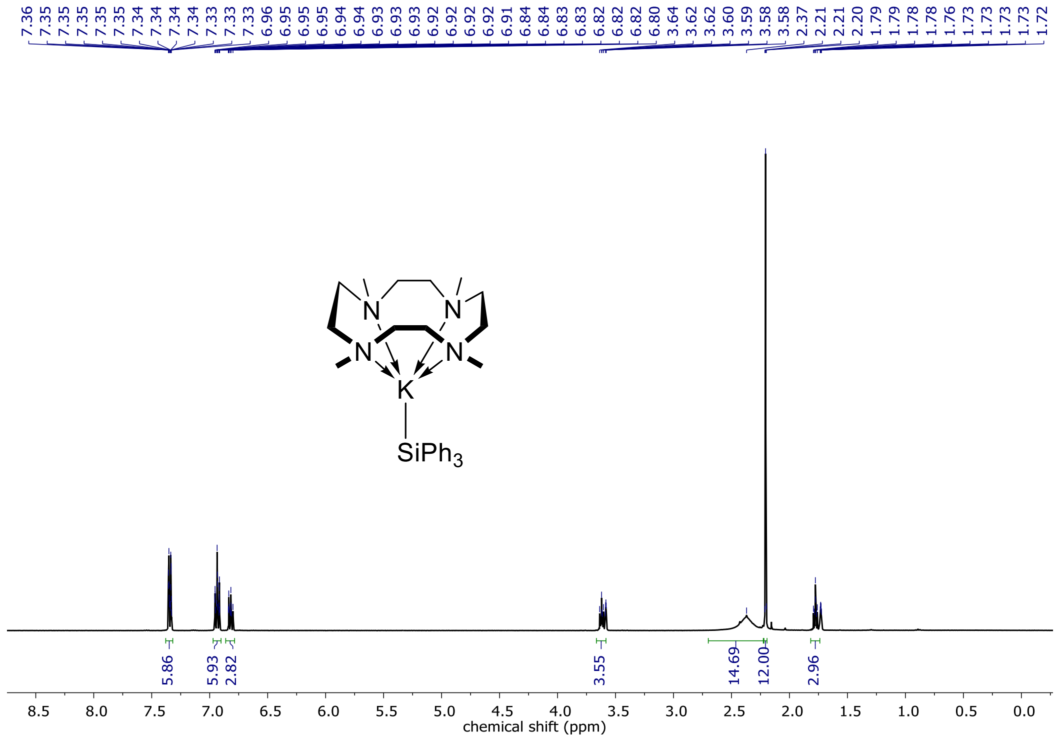

Figure S13. ^{1}H NMR (400 MHz, [D$_8$]THF, 25 °C) of [(Me$_4$TACD)(thf)KSiPh$_3$] (**[THF]-3**).

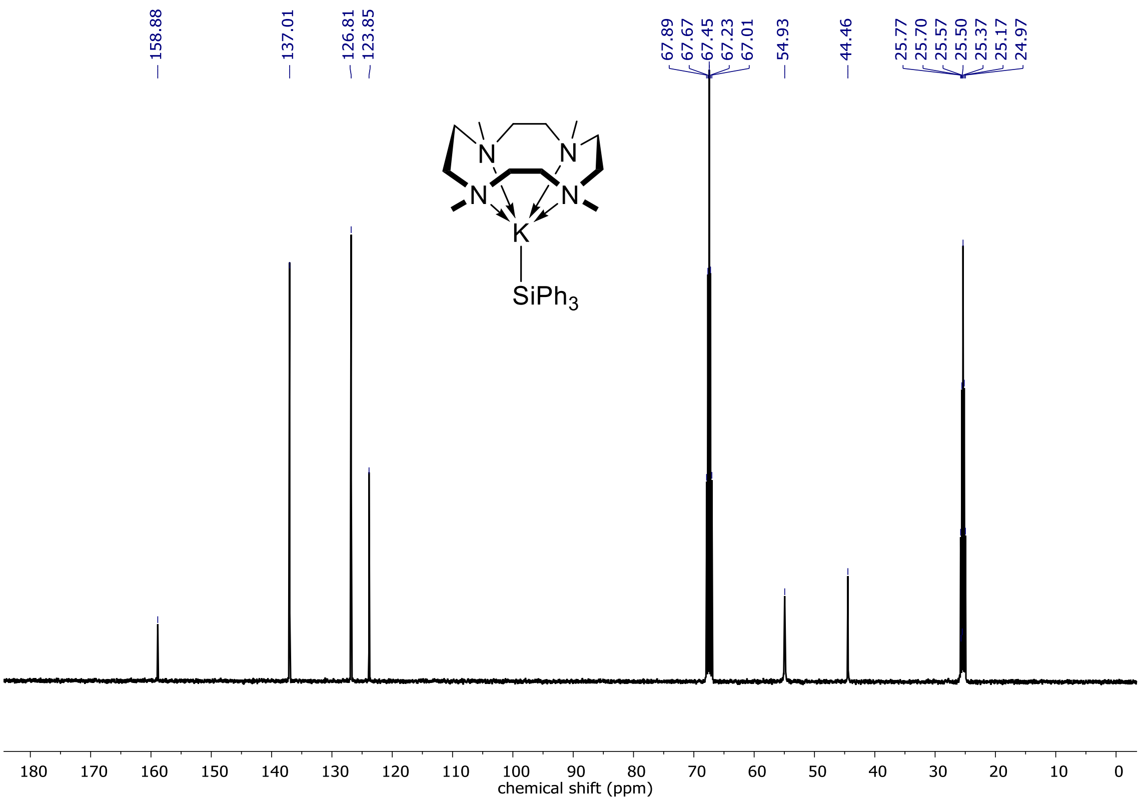

Figure S14. $^{13}C\{^{1}H\}$ NMR (101 MHz, [D_8]THF, 25 °C) of [K(Me_4TACD)$SiPh_3$] (**3**).

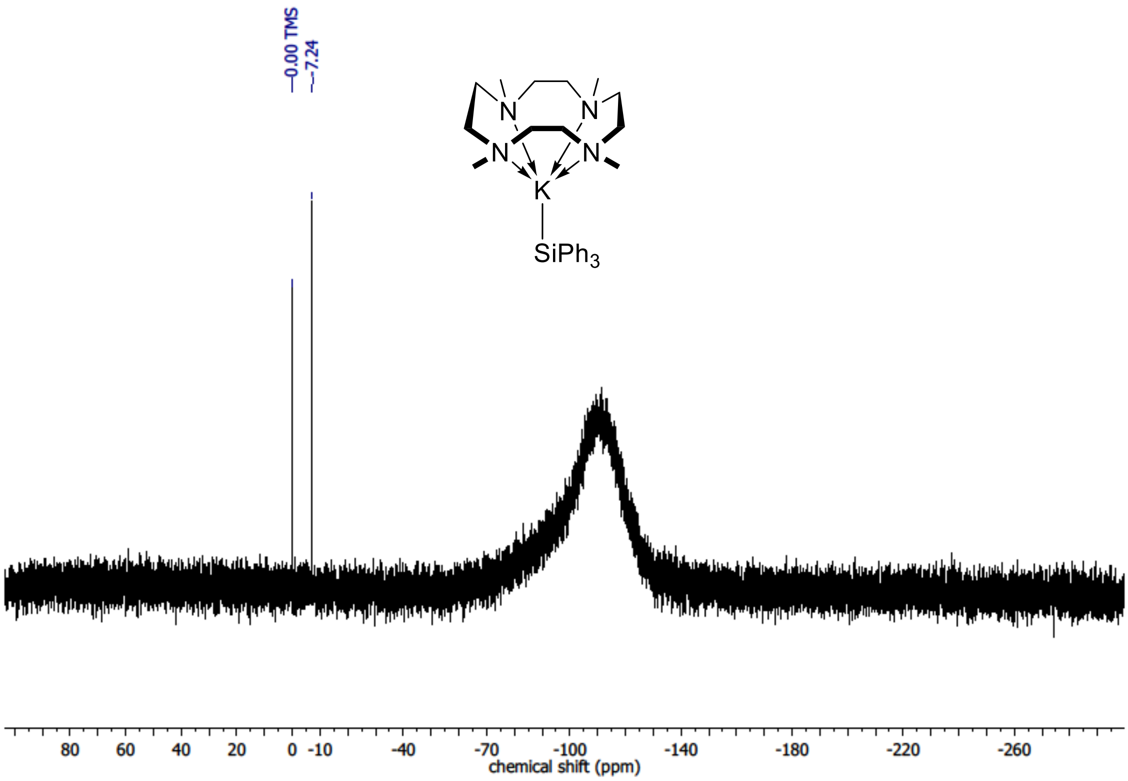

Figure S15. $^{29}Si\{^{1}H\}$ NMR (80 MHz, [D_8]THF, 25 °C) of [K(Me_4TACD)$SiPh_3$] (**3**).

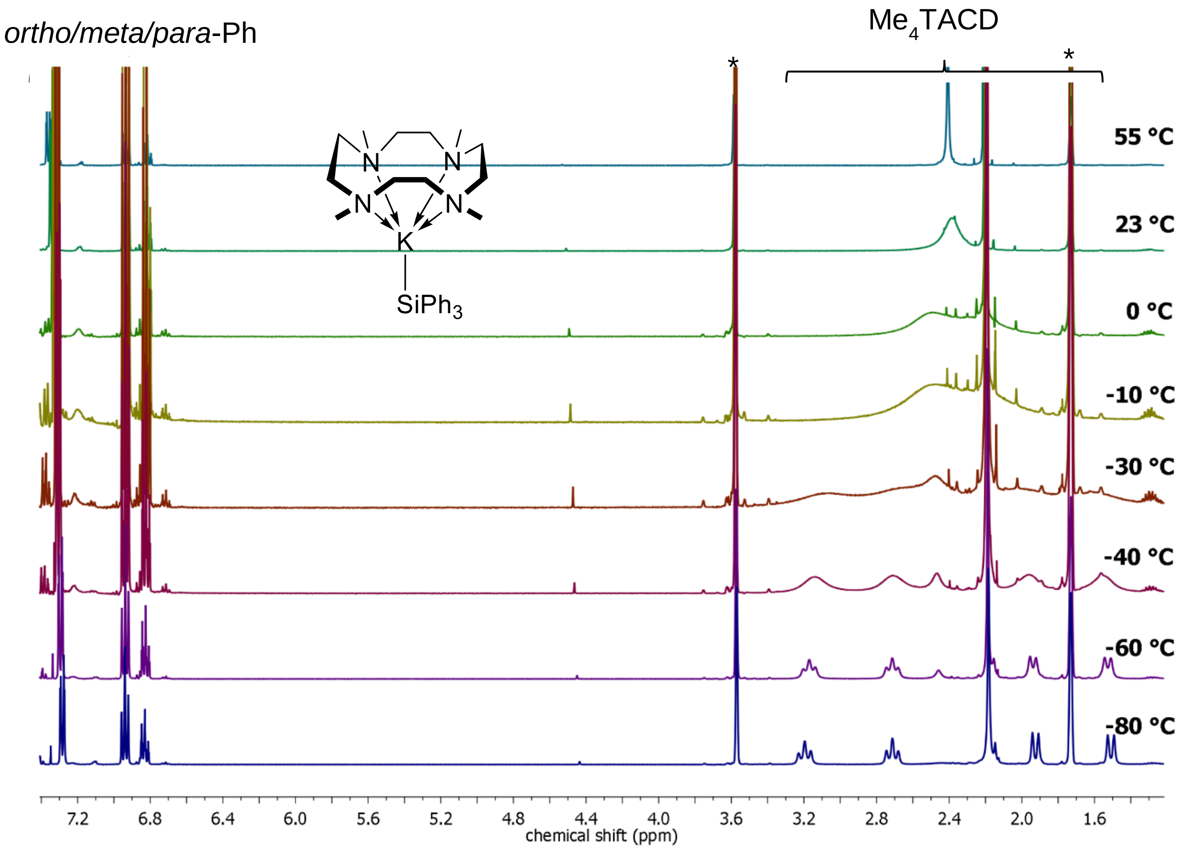

Figure S16. ^{1}H NMR (400 MHz, [D_8]THF of [K(Me_4TACD)$SiPh_3$] (**3**) at indicated temperatures.

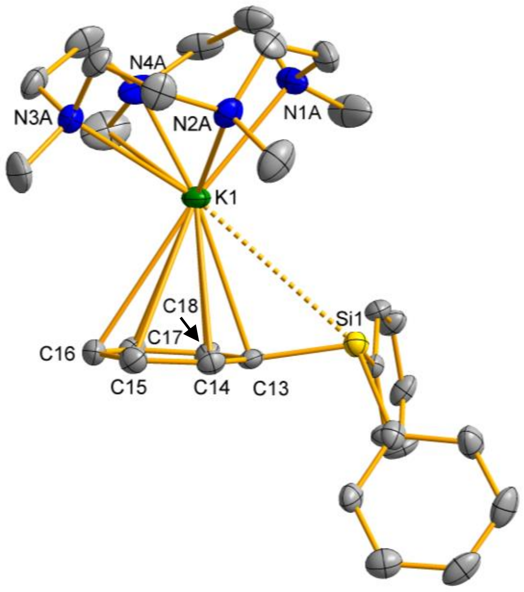

Figure S17. Molecular structures of [(Me_4TACD)KSiPh_3] (**3**). Displacement parameters are shown at a 50% probability level. Hydrogen atoms are omitted for clarity. Selected interatomic distances [Å] and angles [°]: K1-N1A 2.783(5), K1-N2A 2.796(5), K1-N3A 2.863(5), K1-N4A 2.778(6), K1-C13 3.075(3), K1-C14 3.142(3), K1-C17 3.257(4), K1-C18 3.123(3), Si1-C13-C18-C17 166.8(2).

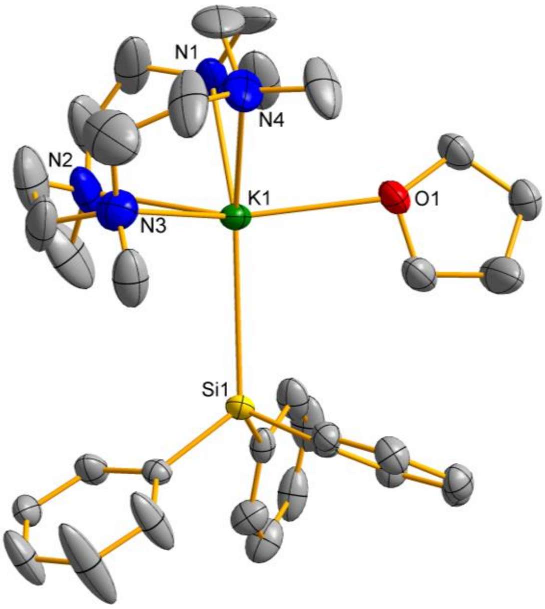

Figure S18. Molecular structures of [(Me_4TACD)(thf)KSiPh_3] (**[THF]-3**). Displacement parameters are shown at a 50% probability level. Hydrogen atoms are omitted for clarity. Selected interatomic distances [Å]: K1-Si1 3.4328(11), K1-O1 2.844(2), K1-N1 2.800(3), K1-N2 2.845(3), K1-N3 2.852(3), K1-N4 2.855(3).

2.7. Synthesis of [(Me$_4$TACD)RbSiPh$_3$]$_2$ (4).

Ph$_3$SiSiMe$_3$ (332 mg, 1.0 mmol) and [RbOtBu] (160 mg, 1.0 mmol) were dissolved in THF (5 mL) in stirred at 25 °C. After 30 min, Me$_4$TACD (232 mg, 1.0 mmol) was added dropwise. After 4 h the solution was reduced *in vacuo* to ca 3 mL, layered with *n*-pentane (4 mL) and stored at –30 °C. After 16 h, orange microcrystals formed. The supernatant was decanted off, the solid washed with n-pentane (3 x 2 mL) and dried *in vacuo* to give [(Me$_4$TACD)RbSiPh$_3$]$_2$ (**2**) (378 mg, 0.33 mmol, 66%) as orange microcrystals.

Single crystals of [(Me$_4$TACD)RbSiPh$_3$]$_2$ (**4**) were grown from a THF/*n*-pentane solution at –30 °C.

^{1}H NMR (400 MHz, [D$_8$]THF, 25 °C): δ = 2.20 (s, 12 H, NC*H*$_3$), 2.25 – 2.55 (br, 16 H, NC*H*$_2$), 6.77 – 6.84 (m, 3 H, *para*-Ph), 6.90 – 6.96 (m, 6 H, *meta*-Ph), 7.36 – 7.33 (m, 6 H, *ortho*-Ph) ppm.

^{13}C{^{1}H} NMR (101 MHz, [D$_8$]THF, 25 °C): δ = 44.34 (CH$_3$), 55.05 (CH$_2$), 123.71 (*para*-Ph), 126.78 (*meta*-Ph), 136.99 (*ortho*-Ph), 159.31 (*ipso*-Ph) ppm.

^{29}Si{^{1}H} NMR (80 MHz, [D$_8$]THF, 25 °C): δ = –5.77 ppm.

Anal. Calcd for $C_{60}H_{86}N_8Rb_2Si_2$ (1146.51 g·mol^{-1}): C, 62.86; H, 7.56; N, 9.77. Found: C, 61.19; H, 7.56; N, 9.77.

2.8. NMR spectra of [(Me$_4$TACD)RbSiPh$_3$]$_2$ (4)

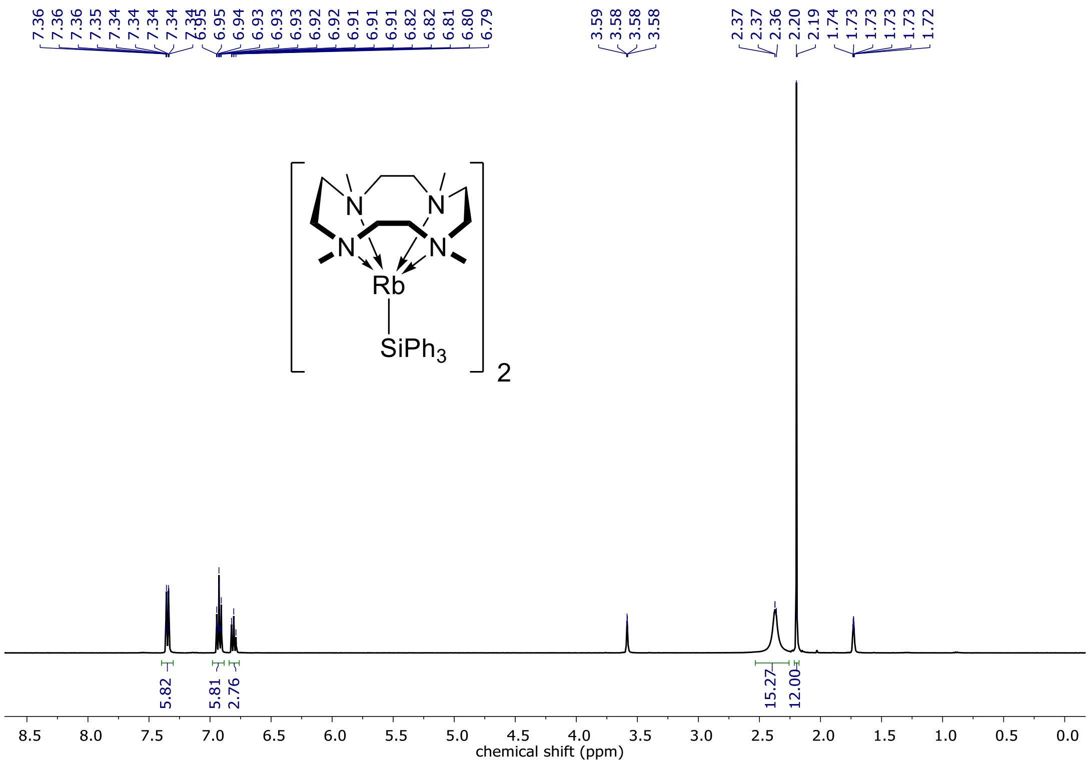

Figure S19. ^{1}H NMR (400 MHz, [D$_8$]THF, 25 °C) of [(Me$_4$TACD)SiPh$_3$]$_2$ (**4**).

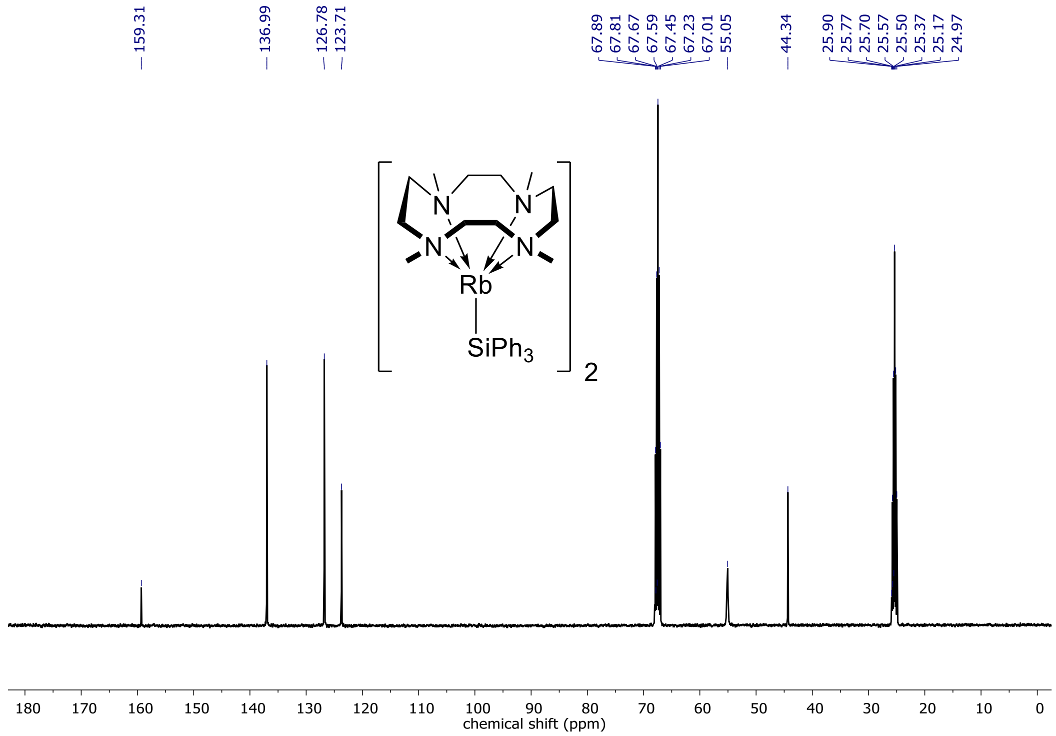

Figure S20. ^{13}C{^{1}H} NMR (101 MHz, [D$_8$]THF, 25 °C) of [(Me$_4$TACD)SiPh$_3$]$_2$ (**4**).

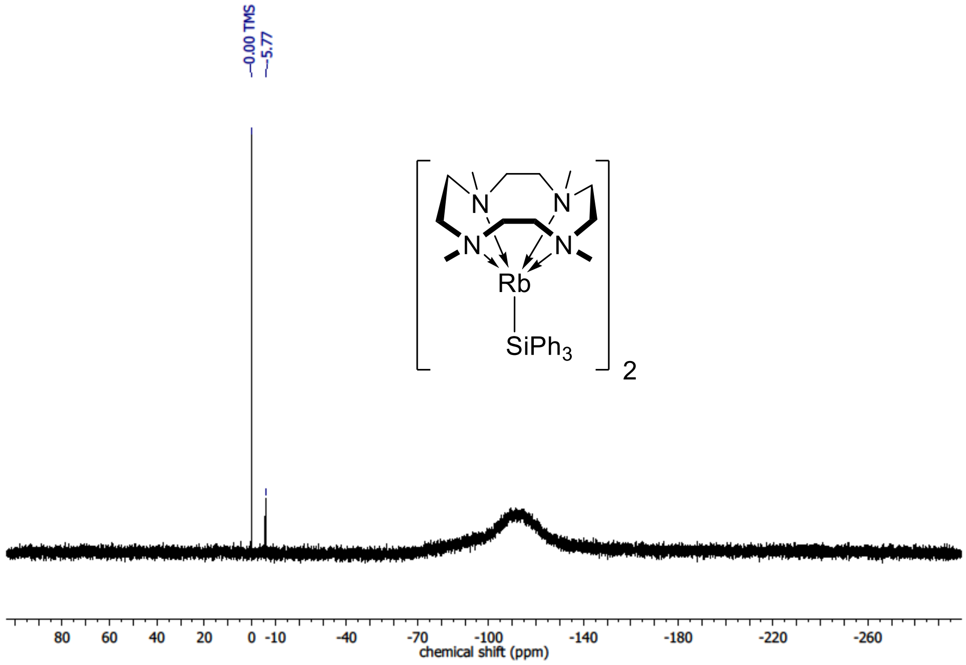

Figure S21. $^{29}Si\{^1H\}$ NMR (80 MHz, $[D_8]THF$, 25 °C) of $[(Me_4TACD)RbSiPh_3]_2$ (**4**).

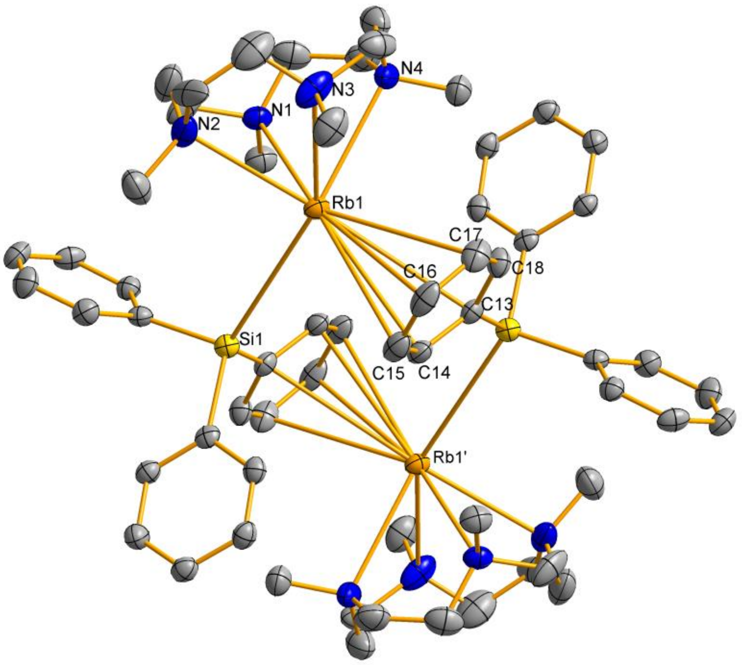

Figure S22. Molecular structures of $[(Me_4TACD)RbSiPh_3]_2$ (**4**). Displacement parameters are shown at a 50% probability level. Hydrogen atoms are omitted for clarity. Selected interatomic distances [Å] and angles [°]: Rb1-Si1 3.775(2), Rb1-N1 2.966(3), Rb1-N2 3.078(4), Rb1-N3 3.095(4), Rb1-N4 3.029(4), Rb1-C13 3.727(4), Rb1-C14 3.488(4), Rb1-C15 3.389(4), Rb1-C16 3.476(4), Rb1-C17 3.648(5), Si1-C13-C14-C15 -171.7(3), Si1-C25-C26-C27 -169.7(3).

2.9. Synthesis of [(Me$_4$TACD)CsSiPh$_3$]$_\infty$ (5)

$Ph_3SiSiMe_3$ (333 mg, 1.0 mmol) and [CsOtBu] (206 mg, 1.0 mmol) were dissolved in THF (10 mL) in stirred at 25 °C. After 30 min, Me$_4$TACD (232 mg, 1.0 mmol) was added dropwise. After 4 h the solution was reduced *in vacuo* to ca 3 mL, layered with *n*-pentane (4 mL) and stored at –30 °C. After 16 h, orange microcrystals formed. The supernatant was decanted off, the solid washed with *n*-pentane (3 x 2 mL) and dried *in vacuo* to give [(Me$_4$TACD)CsSiPh$_3$]$_\infty$ (**5**) (348 mg, 62%) as orange microcrystals.

Single crystals of [(Me$_4$TACD)CsSiPh$_3$]$_\infty$ (**5**) were grown from THF/*n*-pentane at –30 °C.

^{1}H NMR (400 MHz, [D$_8$]THF, 25 °C): δ = 2.18 (s, 12 H, CH_3), 2.25 – 2.55 (br, 16 H, CH_2), 6.77 – 6.86 (m, 3 H, *para*-Ph), 6.90 – 6.98 (m, 6 H, *meta*-Ph), 7.33 – 7.40 (m, 6 H, *ortho*-Ph) ppm.

^{13}C{^{1}H} NMR (101 MHz, [D$_8$]THF, 25 °C): δ = 44.20 (CH_3), 55.15 (CH_2), 123.62 (*para*-Ph), 126.82 (*meta*-Ph), 137.02 (*ortho*-Ph), 159.70 (*ipso*-Ph) ppm.

^{29}Si{^{1}H} NMR (80 MHz, [D$_8$]THF, 25 °C): δ = –3.09 ppm.

Anal. Calcd for [$C_{30}H_{43}N_4CsSi$]$_\infty$ (620.69 g·mol^{-1}): C, 58.05; H, 6.98; N, 9.03. Found: C, 56.61; H, 6.97; N, 9.19%.

2.10. NMR spectra of [(Me$_4$TACD)CsSiPh$_3$]$_\infty$ (5)

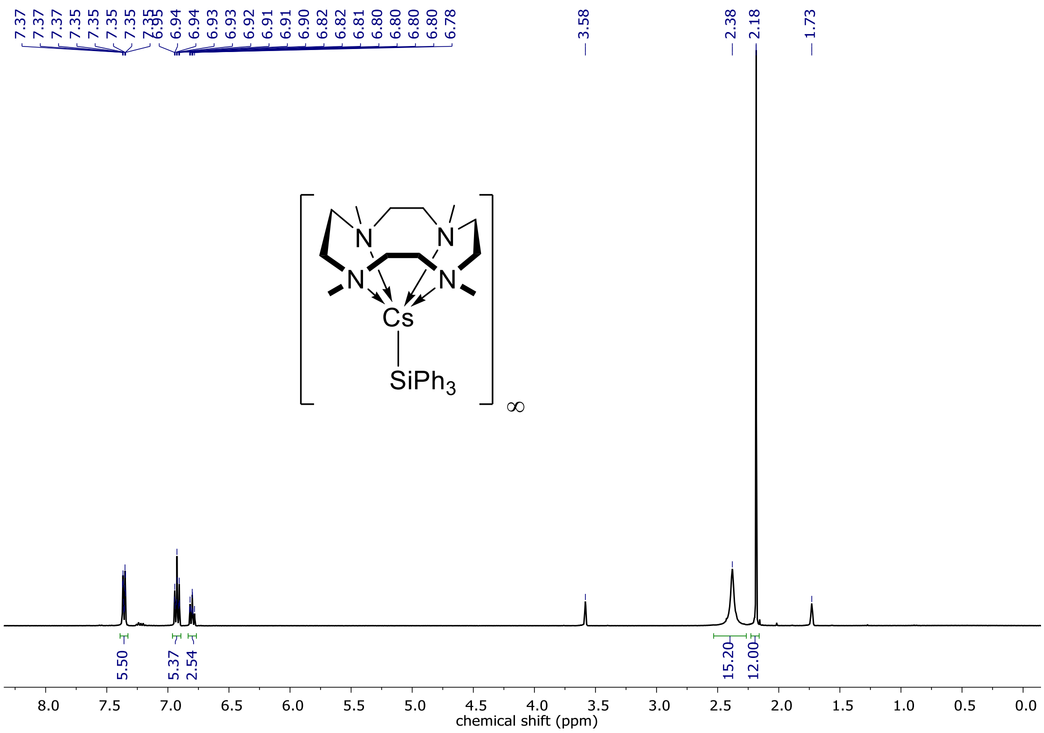

Figure S23. ^{1}H NMR (400 MHz, [D$_8$]THF, 25 °C) of [(Me$_4$TACD)CsSiPh$_3$]$_\infty$ (**5**).

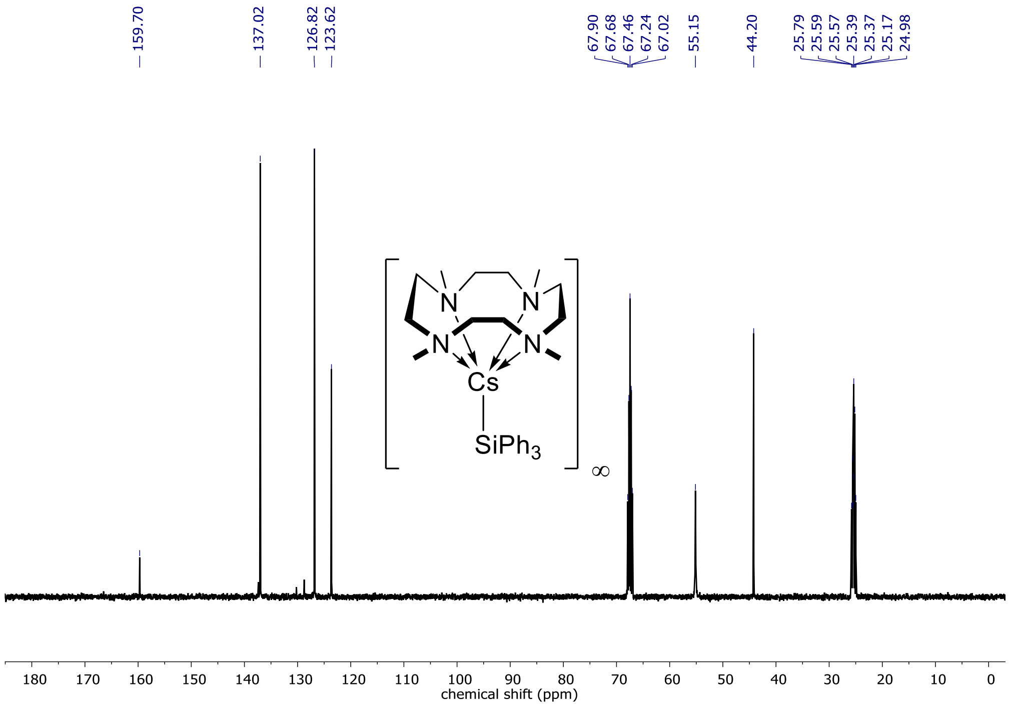

Figure S24. ^{13}C{^{1}H} NMR (101 MHz, [D$_8$]THF, 25 °C) of [(Me$_4$TACD)CsSiPh$_3$]$_\infty$ (**5**).

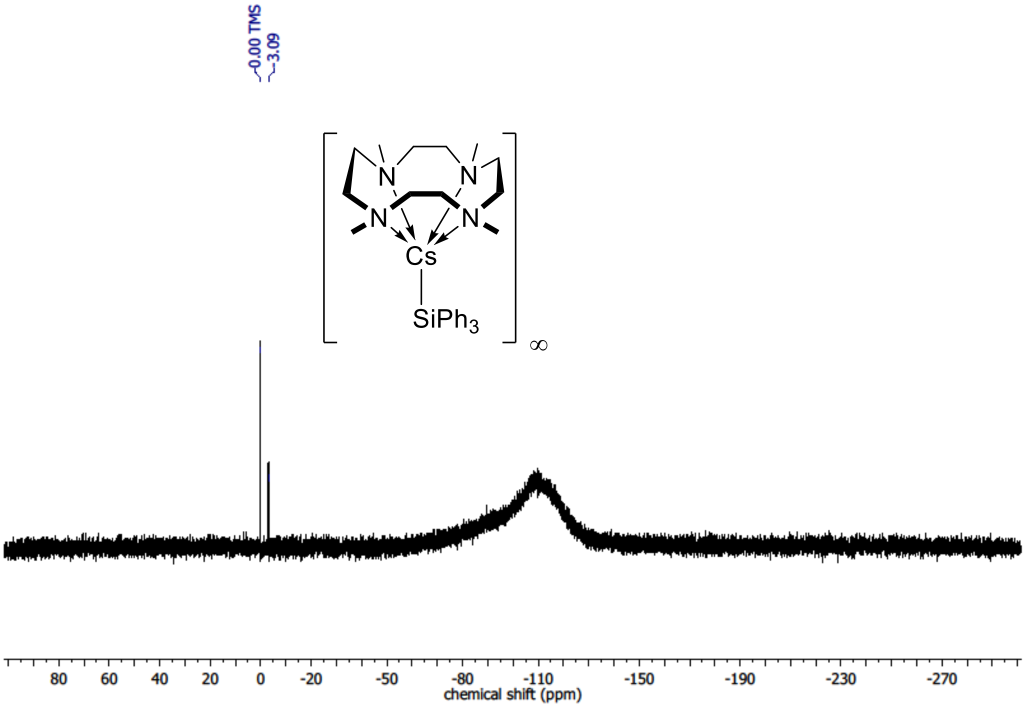

Figure S25. $^{29}Si\{^{1}H\}$ NMR (80 MHz, $[D_8]THF$, 25 °C) of $[(Me_4TACD)CsSiPh_3]_\infty$ (**5**).

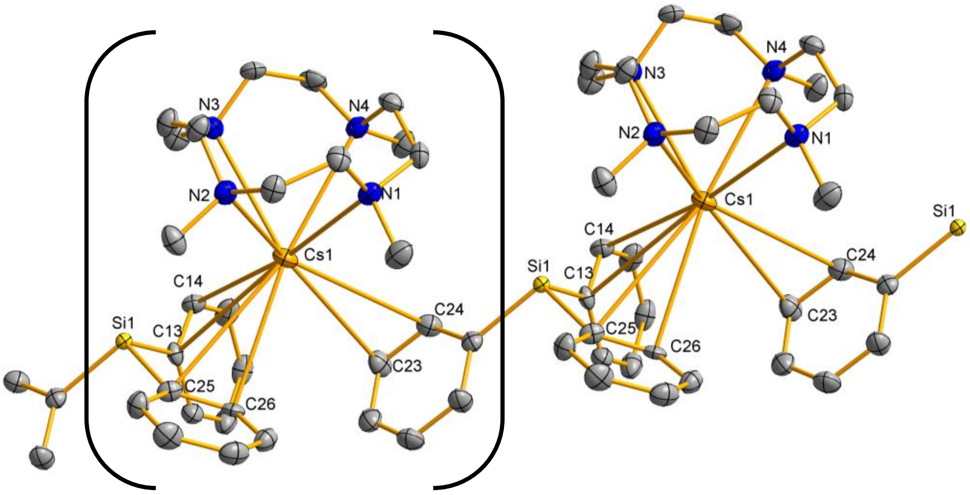

Figure S26. Molecular structures of $[(Me_4TACD)CsSiPh_3]_\infty$ (**5**). Displacement parameters are shown at a 50% probability level. Hydrogen atoms are omitted for clarity. Selected interatomic distances [Å] and angles [°]: Cs1–Si1 3.859(2), Cs1–N1 3.122(7), Cs1–N2 3.197(6), Cs1–N3 3.128(6), Cs1–N4 3.181(8), Cs1–C13 3.770(8), Cs1–C14 3.674(8), Cs1–C23 3.718(11), Cs1–C24 3.817(8), Cs1–C25 3.756(8), Cs1–C26 3.420(8), Si1-C25-C30-C29 -173.8(7).

2.11. Synthesis of [Li(Me_4TACD)SiH_3] (6)

[Li(Me_4TACD)$SiPh_3$] (**1**) (100 mg, 0.20 mmol) was dissolved in benzene (2 mL) and $PhSiH_3$ (65 mg, 0.60 mmol, 3 eq.) was added dropwise at 25 °C. The solution was filtered and layered with *n*-pentane (1 mL). After 2 h, an colorless precipitate formed. The supernatant was decanted off, the precipitation washed with n-pentane (2 x 1 mL) and dried *in vacuo* to give [Li(Me_4TACD)SiH_3] (33 mg, 0.12 mmol, 60 %) as a colorless powder.

^{1}H NMR (400 MHz, [D_8]THF, 25 °C): δ = 1.42 (s, $^1J_{Si\text{-}H}$ = 91.1 Hz, 3 H, SiH_3), 2.37 – 2.29 (m, 8 H, NCH_2), 2.38 (s, 12 H, NCH_3), 2.67 – 2.58 (m, 8 H, NCH_2) ppm.

^{13}C{^{1}H} NMR (101 MHz, [D_8]THF, 25 °C): δ = 45.23 (CH_3), 54.71 (CH_2) ppm

^{29}Si{^{1}H} NMR (80 MHz, [D_8]THF, 25 °C): δ = –160.56 ppm

^{7}Li{^{1}H} NMR (156 MHz, [D_8]THF, 25 °C): δ = 1.50 ppm.

Anal. Calcd for [$C_{12}H_{31}N_4LiSi$]$_\infty$ (266.43 g·mol^{-1}): C, 54.10; H, 11.73; N, 21.03. Found: C, 61.70; H, 11.34; N, 18.61%.

2.12. NMR spectra of [(Me$_4$TACD)LiSiH$_3$] (6)

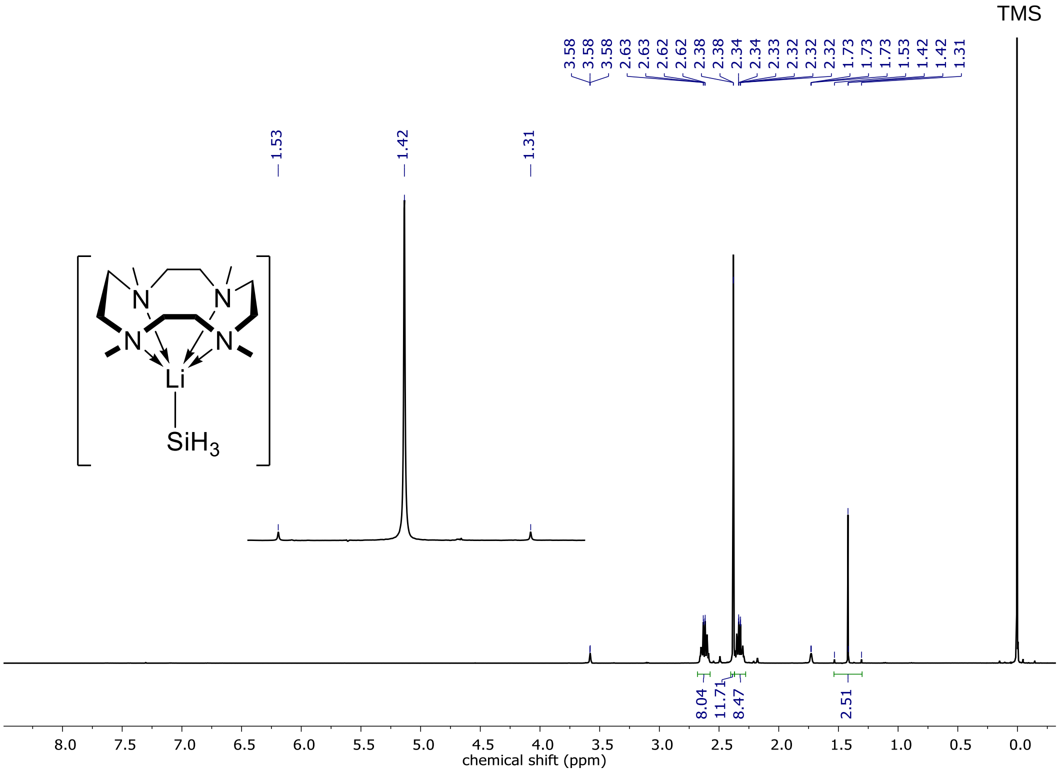

Figure S27. ^{1}H NMR (400 MHz, [D$_8$]THF, 25 °C) of [(Me$_4$TACD)LiSiH$_3$] (**6**).

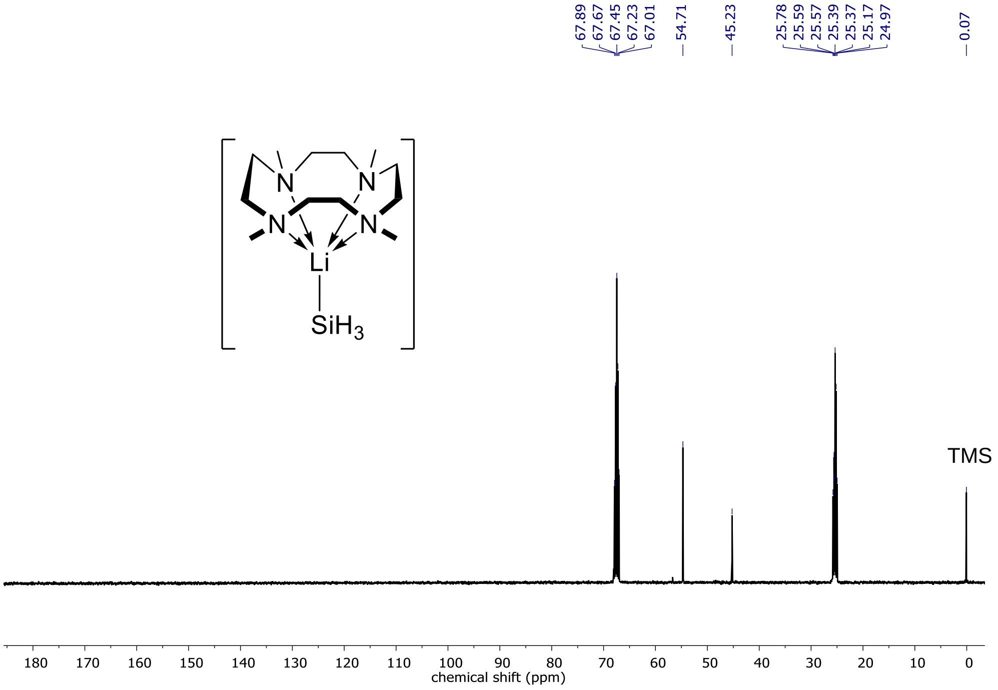

Figure S28. ^{13}C{^{1}H} NMR (101 MHz, [D$_8$]THF, 25 °C) of [(Me$_4$TACD)LiSiH$_3$] (**6**).

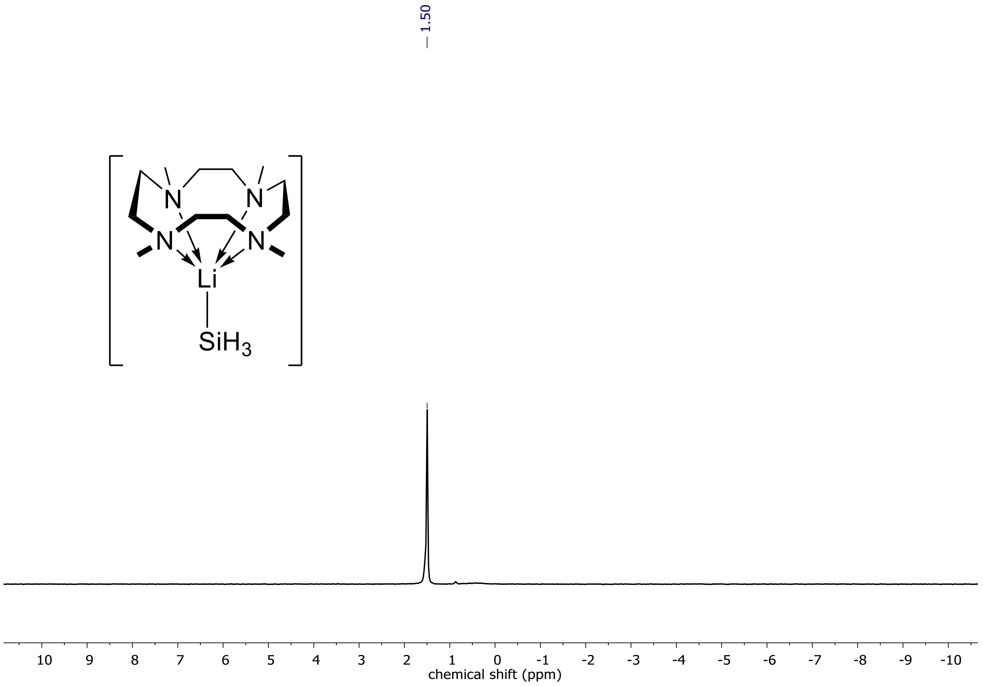

Figure S29. $^{7}Li\{^{1}H\}$ NMR (156 MHz, [D$_8$]THF, 25 °C) of [(Me$_4$TACD)LiSiH$_3$] (**6**).

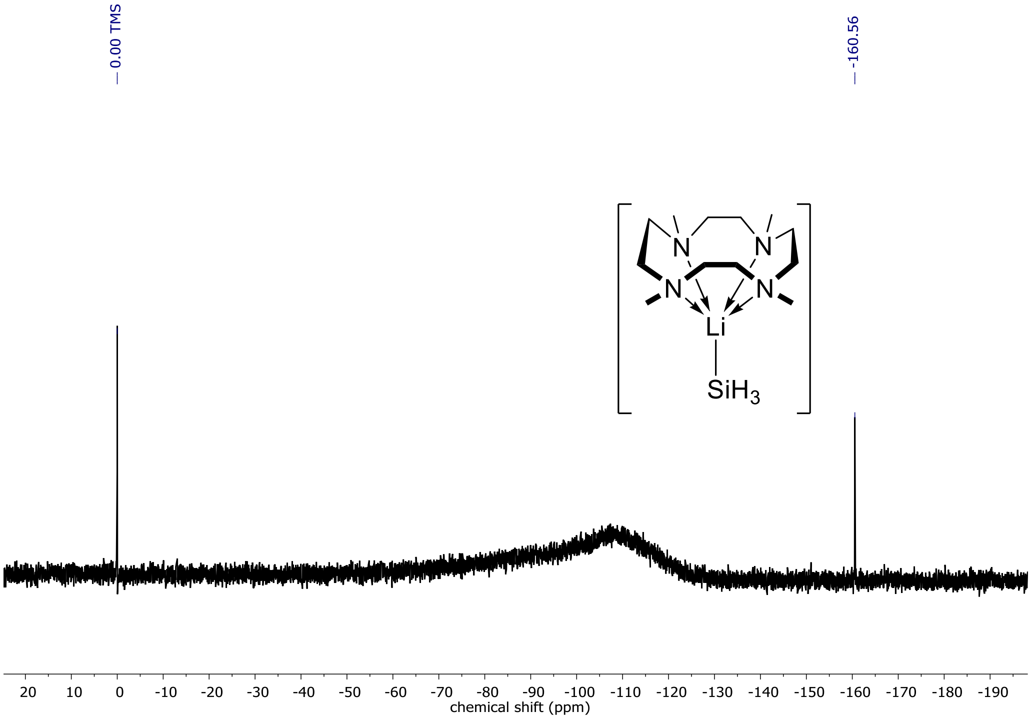

Figure S30. $^{29}Si\{^{1}H\}$ NMR (80 MHz, [D$_8$]THF, 25 °C) of [(Me$_4$TACD)LiSiH$_3$] (**6**).

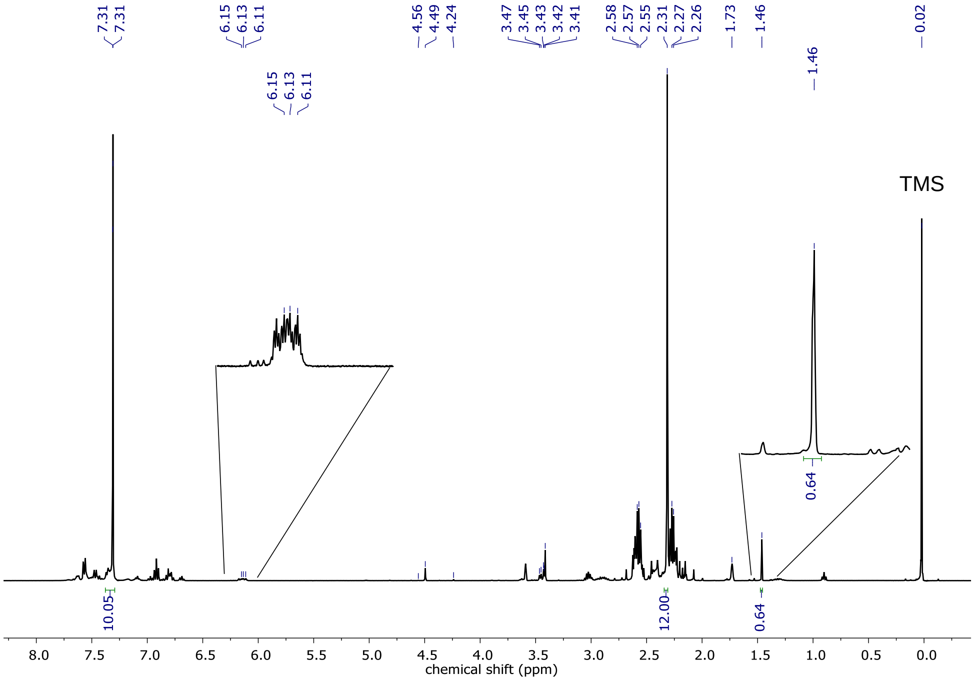

Figure S31. ^{1}H NMR (400 MHz, [D$_8$]THF, 25 °C) of the hydrogenolysis of **1** after 3 d.

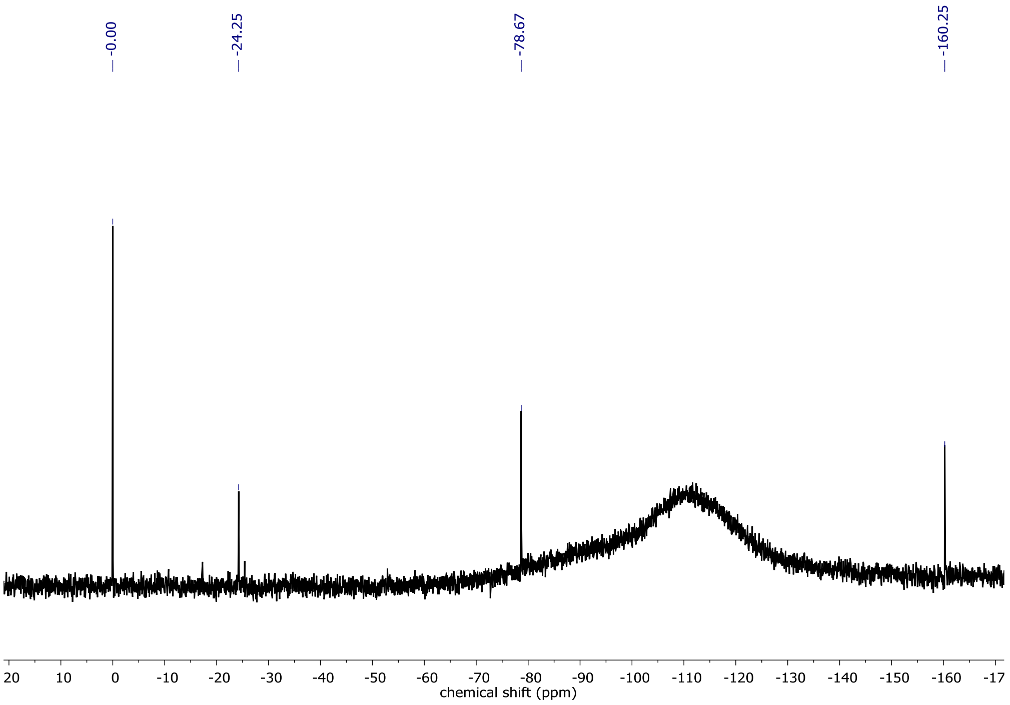

Figure S32. ^{29}Si{^{1}H} NMR (80 MHz, [D$_8$]THF, 25 °C) of the hydrogenolysis of **1** after 3 d.

2.13. Synthesis of [(Me_4TACD)NaSiH_3] (7)

Method A. A degassed solution of [(Me_4TACD)NaSiPh_3] (**2**) (150 mg, 0.29 mmol) in THF (5 mL) was charged with H_2 (1 bar) in a glass autoclave and stirred at 25 °C. After 7 d, the reaction mixture was filtered, layered with *n*-pentane (10 mL) and stored at −30 °C. After 16 h, a colorless precipitate formed. The supernatant was decanted off, the solid was washed with *n*-pentane (3×5 mL) and the solvents were removed under reduced pressure to give [(Me_4TACD)NaSiH_3] (**7**) (78 mg, 0.28 mmol, 93%) as a colorless powder.

Method B. [(Me_4TACD)NaSiPh_3] (**2**) (100 mg, 0.2 mmol) was dissolved in benzene (3 mL) and PhSiH_3 (65 mg, 0.6 mmol) was added dropwise. The solution was filtered and stored at room temperature. After 16 h, colorless crystals formed. The supernatant was decanted off, the crystals were washed with *n*-pentane (2 x 1 mL) and dried *in vacuo* to give [(Me_4TACD)NaSiH_3] (**7**) (48 mg, 0.17 mmol, 85%) as colorless crystals.

Single crystals of [(Me_4TACD)NaSiH_3] (**7**) were grown by layering a solution of **2** in benzene with a solution of PhSiH_3 in benzene.

^{1}H NMR (400 MHz, [D_8]THF, 25 °C): δ = 1.26 (s, $^{1}J_{Si\text{-}H}$ = 85.3 Hz, 3 H, SiH_3), 2.37 – 2.22 (m, 20 H, NCH_3 + NCH_2), 2.66 – 2.54 (m, 8 H, NCH_2) ppm.

^{13}C{^{1}H} NMR (101 MHz, [D_8]THF, 25 °C): δ = 44.34 (CH_3), 54.37 (CH_2) ppm

^{29}Si{^{1}H} NMR (80 MHz, [D_8]THF, 25 °C): δ = –172.1 ppm

Anal. calc. for $C_{12}H_{31}N_4NaSi$ (282.48 g mol^{-1}): C, 51.02; H, 11.06; N, 19.83. Found: C, 49.44; H, 10.37; N, 19.06 %.

2.14. NMR spectra of [(Me$_4$TACD)NaSiH$_3$] (7)

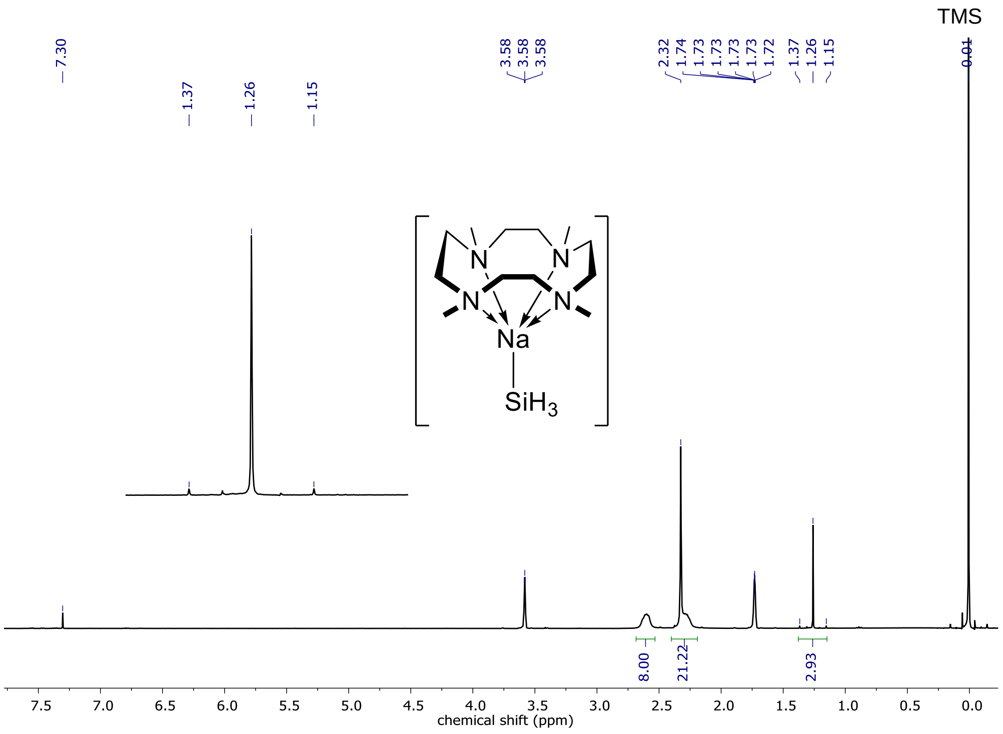

Figure S33. ^{1}H NMR (400 MHz, [D$_8$]THF, 25 °C) of [(Me$_4$TACD)NaSiH$_3$] (**7**).

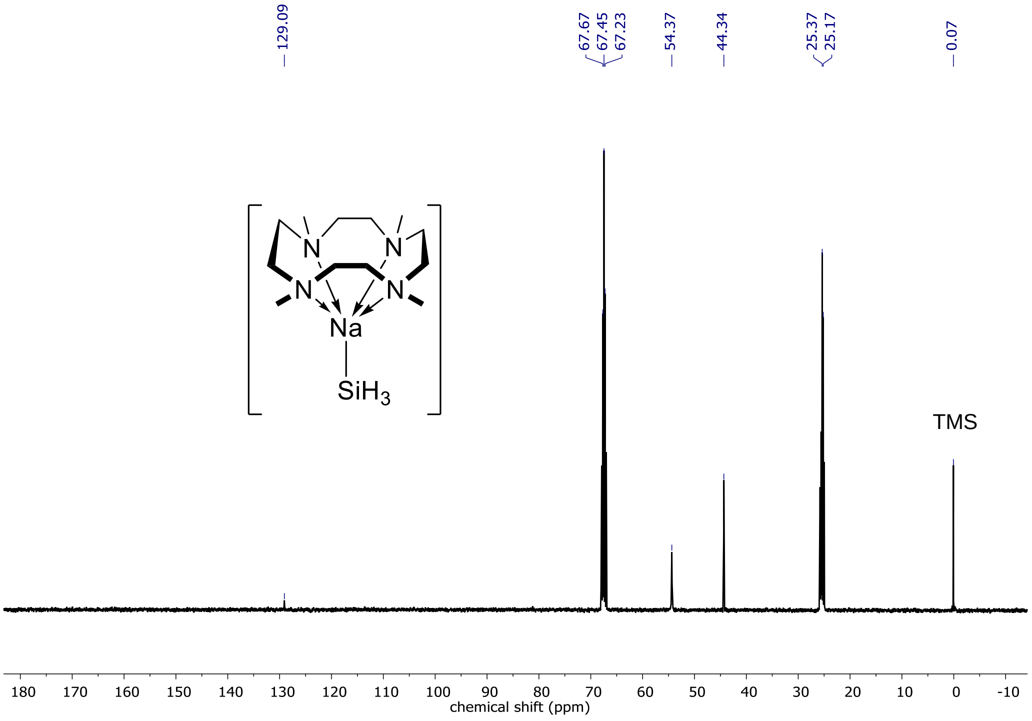

Figure S34. $^{13}C\{^{1}H\}$ NMR (101 MHz, [D$_8$]THF, 25 °C) of [(Me$_4$TACD)NaSiH$_3$] (**7**).

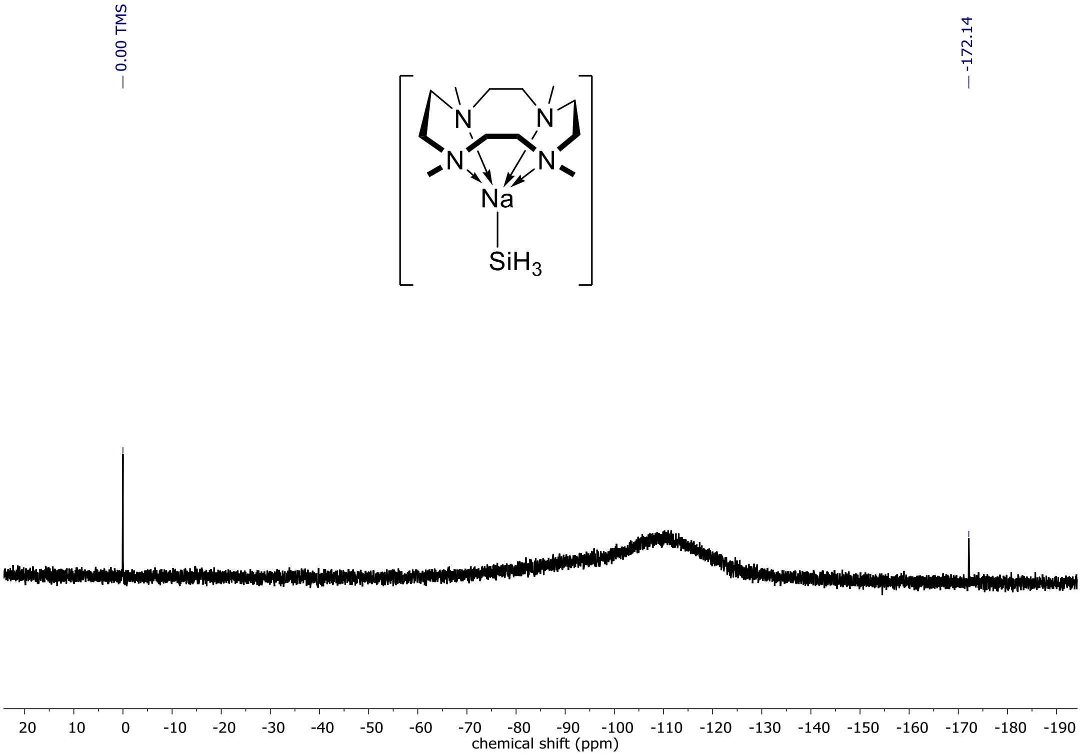

Figure S35. $^{29}Si\{^1H\}$ NMR (80 MHz, [D8]THF, 25 °C) of $[(Me_4TACD)NaSiH_3]$ (**7**).

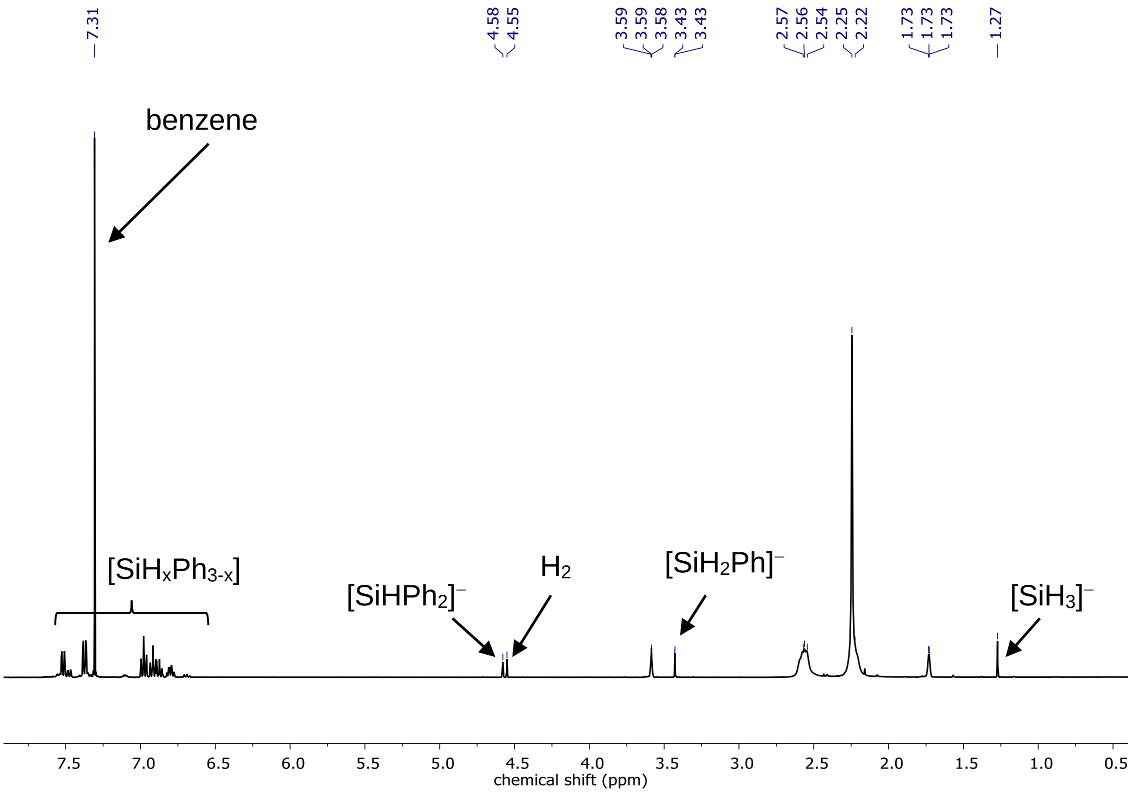

Figure S36. ^1H NMR (400 MHz, [D8]THF, 25 °C) of the hydrogenolysis of **2** after 3 d.

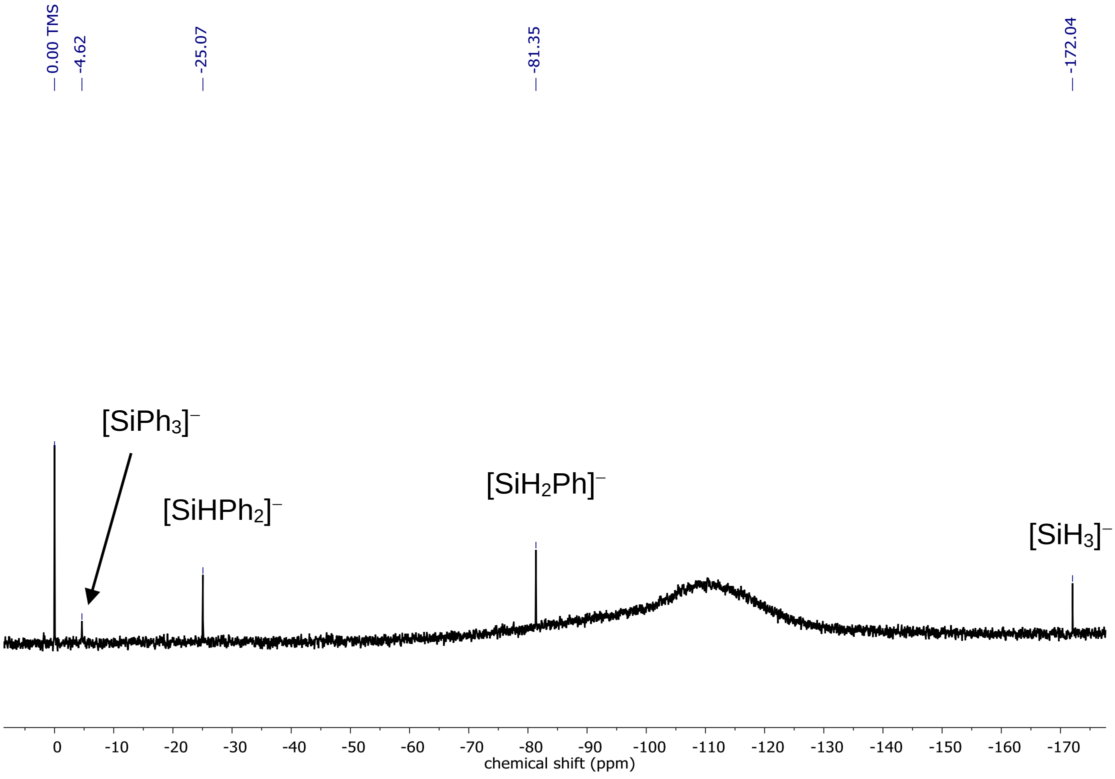

Figure S37. ^{29}Si{^{1}H} NMR (80 MHz, [D_8]THF, 25 °C) of the hydrogenolysis of **2** after 3 d.

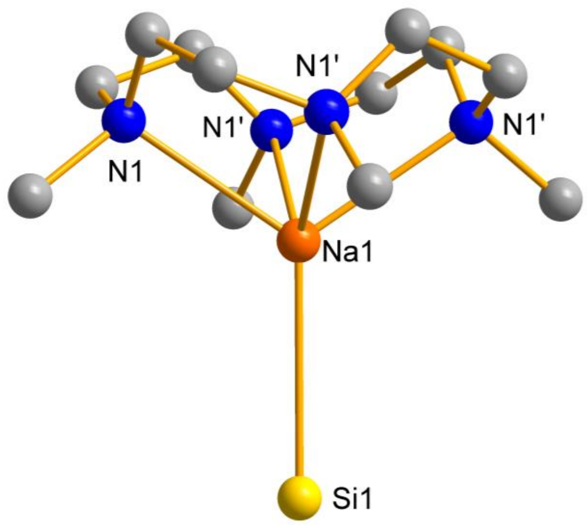

Figure S38. Molecular structures of [(Me_4TACD)NaSiH_3] (**7**). Ball and Stick model. Hydrogen atoms are omitted for clarity.

2.15. Synthesis of $[(Me_4TACD)KSiH_3]_2$ (8)

Method A

A degassed solution of $[(Me_4TACD)KSiPh_3]$ (**3**) (263 mg, 0.50 mmol) in THF (3 mL) was charged with H_2 (1 bar) in a glass autoclave and stirred for 7 d at 25 °C. The reaction mixture was filtered, layered with *n*-pentane (10 mL) and stored at –30 °C. After 16 h, a colorless precipitate formed. The supernatant was decanted off, the solid was washed with *n*-pentane (3×5 mL) and the solvents were removed under reduced pressure to give $[(Me_4TACD)KSiH_3]_2$ (**8**) (120 mg, 0.20 mmol, 81 %) was isolated as an colorless powder.

Method B

$[(Me_4TACD)KSiPh_3]$ (**3**) (100 mg, 0.19 mmol) was dissolved in benzene (3 mL) and a $PhSiH_3$ (65 mg, 0.6 mmol) was added. The solution was filtered and stored at room temperature. After 16 h, colorless crystals formed. The supernatant was decanted off, the crystals were washed with n-pentane (2 x 1 mL) and dried *in vacuo* to give $[(Me_4TACD)KSiH_3]_2$ (**8**) (42 mg, 0.07 mmol, 74%) as colorless crystals.

Single crystals of $[(Me_4TACD)KSiH_3]_2$ (**8**) were grown by layering a solution of **3** in benzene with a solution of $PhSiH_3$ in benzene.

^{1}H NMR (400 MHz, $[D_8]$THF, 25 °C): δ = 1.28 (s, 3H, $^1J_{Si\text{-}H}$ = 76 Hz, SiH_3), 2.33 (s, 12H, NCH_3), 2.34 – 2.55 (br, 16H, NCH_2) ppm.

^{13}C{^{1}H} NMR (100.6 MHz, $[D_8]$THF, 25 °C): δ = 44.84 (CH_3), 54.92 (CH_2) ppm.

^{29}Si{^{1}H} NMR (80 MHz, $[D_8]$THF, 25 °C): δ = –166.7 ppm.

Anal. calc. for $C_{24}H_{61}N_8K_2Si_2$ (597.18 g mol^{-1}): C, 48.27; H, 10.46; N, 18.76. Found: C, 47.71; H, 10.15; N, 18.32.

2.16. NMR spectra of [(Me$_4$TACD)KSiH$_3$]$_2$

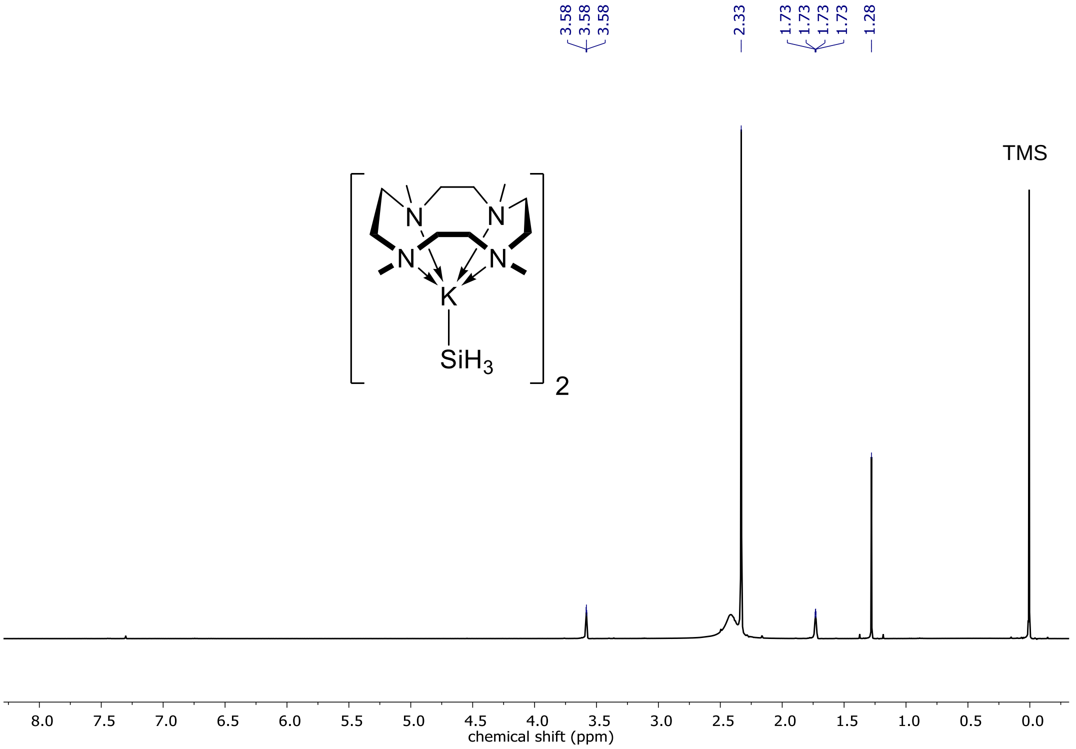

Figure S39. ^{1}H NMR (400 MHz, [D$_8$]THF, 25 °C) of [(Me$_4$TACD)KSiH$_3$]$_2$ (**8**).

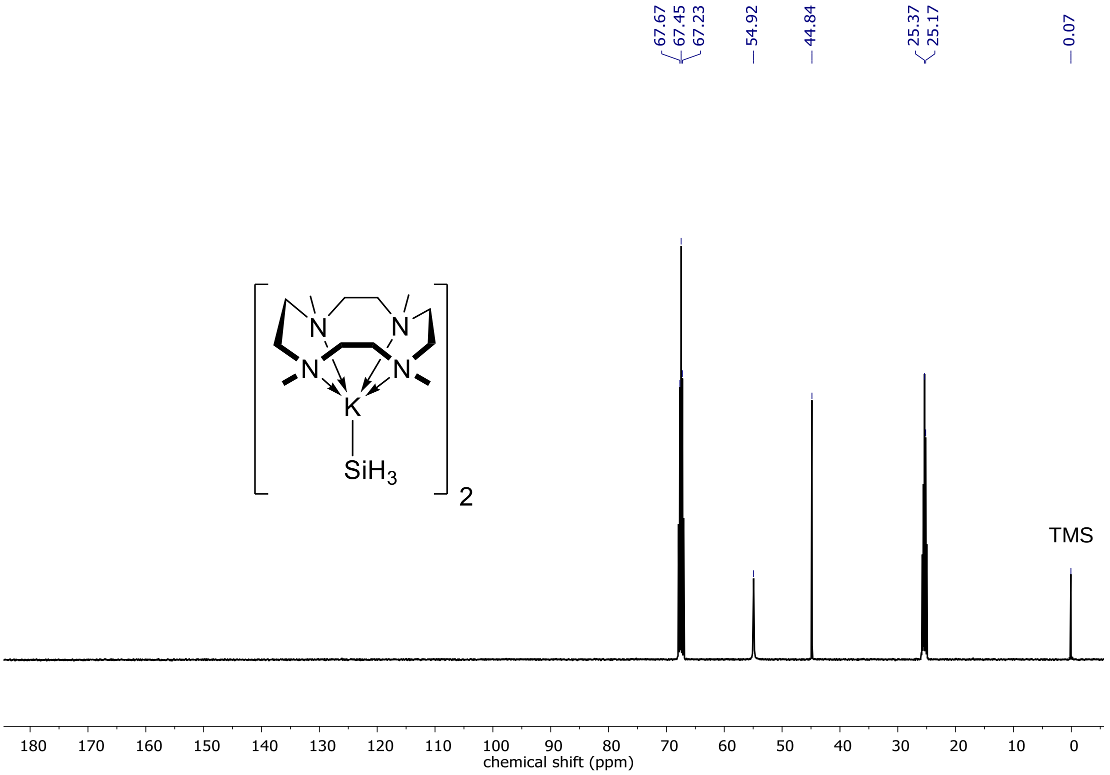

Figure S40. ^{13}C{^{1}H} NMR (101 MHz, [D$_8$]THF, 25 °C) of [(Me$_4$TACD)KSiH$_3$]$_2$ (**8**).

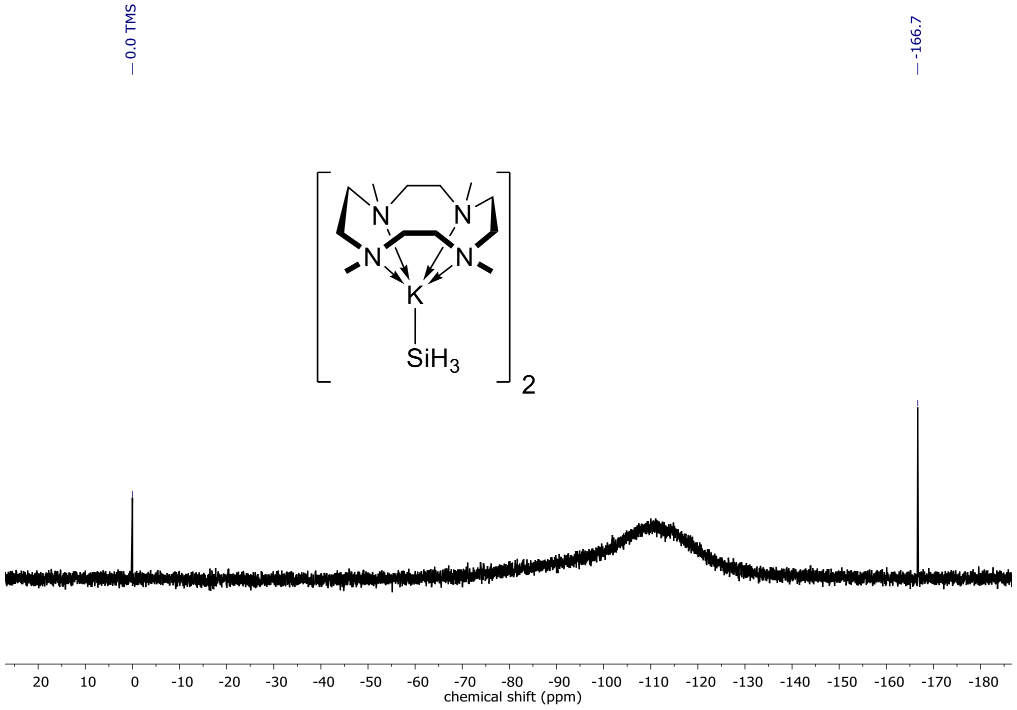

Figure S41. $^{29}Si\{^{1}H\}$ NMR (80 MHz, $[D_8]THF$, 25 °C) of $[(Me_4TACD)KSiH_3]_2$ (**8**).

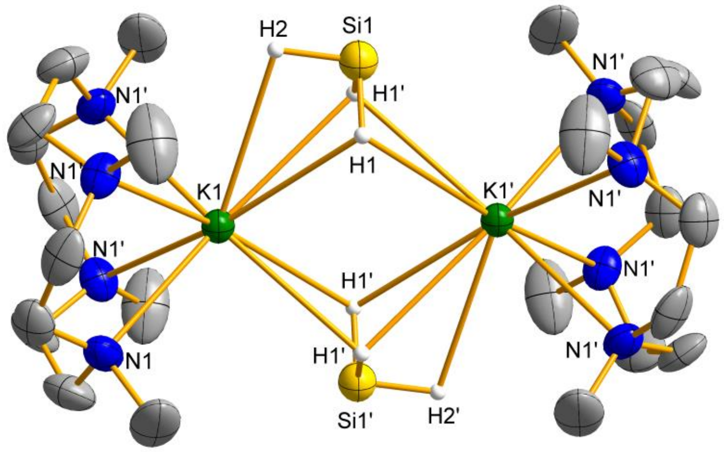

Figure S42. Molecular structures of $[(Me_4TACD)KSiH_3]_2$ (**8**). Displacement parameters are shown at a 50% probability level. Hydrogen atoms and lattice benzene molecules are omitted for clarity. Selected interatomic distances [Å]: K1–K1‘ 4.4691(17), K1–Si1 3.5625(13), K1–Si1 3.5625(13), K1–H1 3.06(2), K1–N1 2.862(2), Si1–H1 1.31(3), Si1–H2 1.32(7)

2.17. Synthesis of $[(Me_4TACD)RbSiH_3]_2$ (9)

$[(Me_4TACD)RbSiPh_3]_2$ (**4**) (57 mg, 0.05 mmol) was dissolved in benzene (2 mL) and a $PhSiH_3$ (32 mg, 0.6 mmol) was added, leading to a colorless crystalline precipitate. The supernatant was decanted off, the microcrystals were washed with n-pentane (2 x 1 mL) and dried *in vacuo*, to give $[(Me_4TACD)RbSiH_3]_2$ (**9**) (24 mg, 35 µmol, 70 %) as colorless microcrystals.

Single crystals of $[(Me_4TACD)RbSiH_3]_2$ (**9**) were grown by layering a solution of **4** in benzene with a solution of $PhSiH_3$ in benzene.

^1H NMR (400 MHz, $[D_8]$THF, 25 °C): δ = 1.36 (s, $^1J_{Si\text{-}H}$ = 75 Hz, 3 H, SiH_3), 2.30 (s, 12 H, CH_3), 2.35 – 2.55 (br, 16 H, CH_2) ppm.

$^{13}C\{^1H\}$ NMR (101 MHz, $[D_8]$THF, 25 °C): δ = 44.64 (CH_3), 55.17 (CH_2) ppm.

$^{29}Si\{^1H\}$ NMR (80 MHz, $[D_8]$THF, 25 °C): δ = –164.0 ppm.

Anal. calc. for $C_{24}H_{61}N_8Rb_2Si_2{\bullet}C_6H_6$ (768.04 g mol^{-1}): C, 46.92; H, 8.92; N, 14.59. Found: C, 40.40; H, 7.92; N, 14.89%.

2.18. NMR spectra of [(Me$_4$TACD)RbSiH$_3$]$_2$ (9)

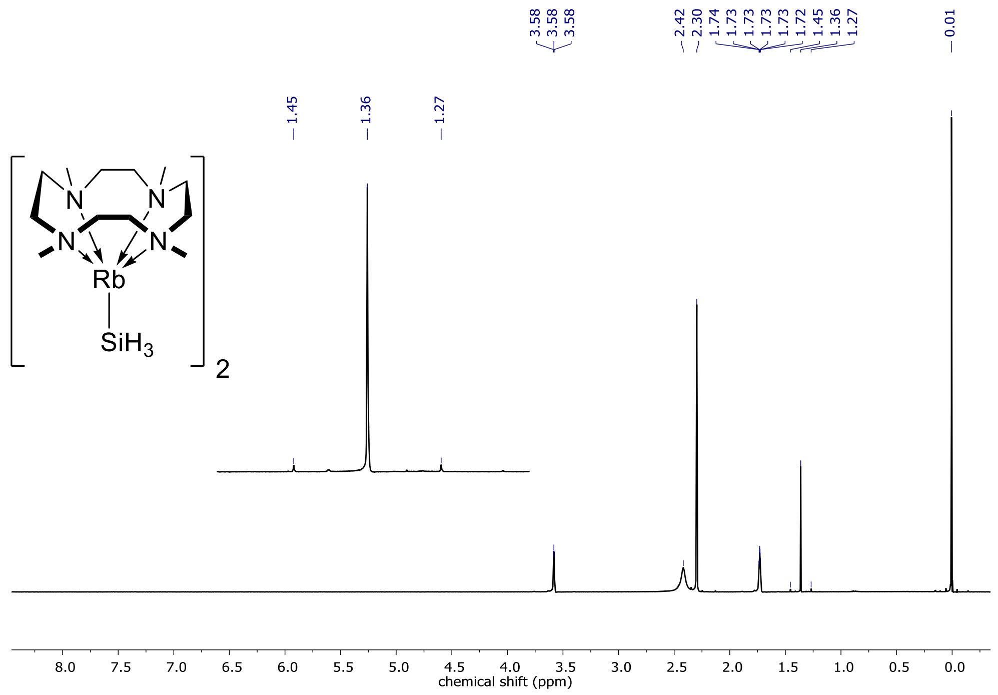

Figure S43. ^{1}H NMR (400 MHz, [D$_8$]THF, 25 °C) of [(Me$_4$TACD)RbSiH$_3$]$_2$ (**9**).

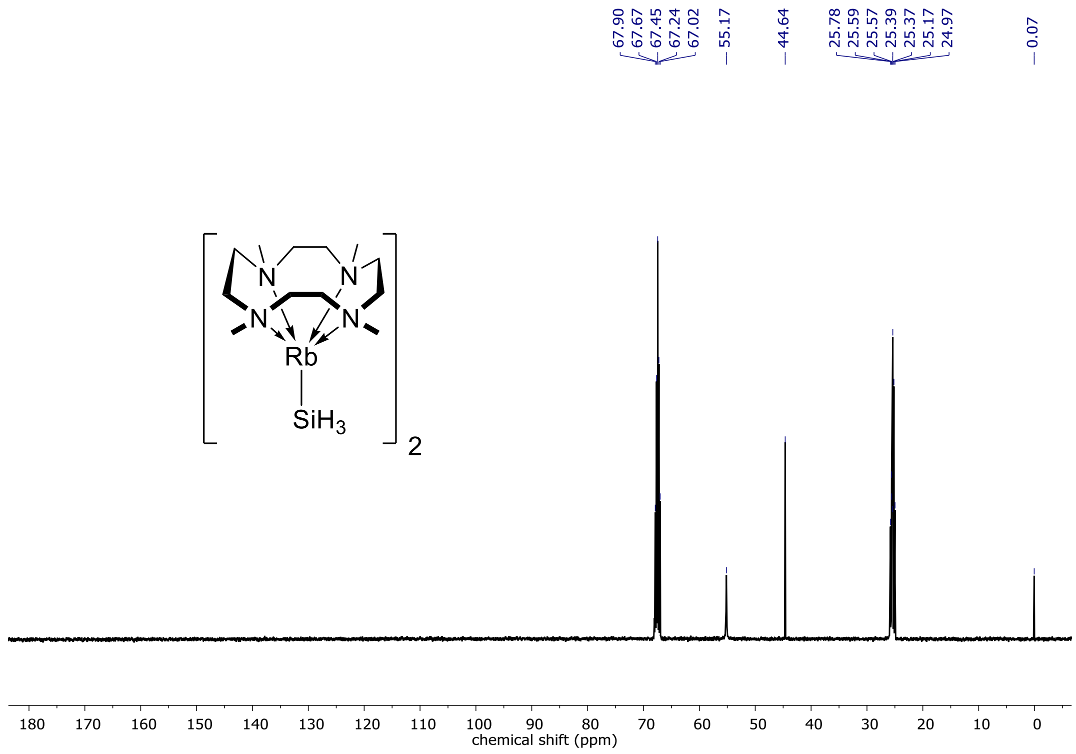

Figure S44. ^{13}C{^{1}H} NMR (101 MHz, [D$_8$]THF, 25 °C) of [(Me$_4$TACD)RbSiH$_3$]$_2$ (**9**).

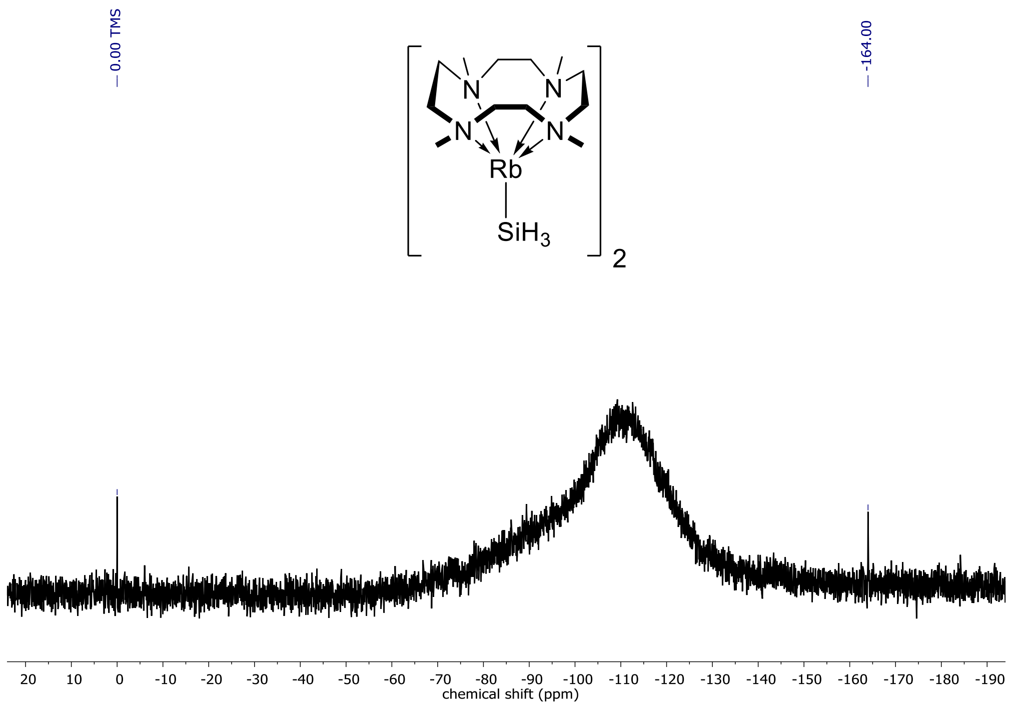

Figure S45. $^{29}Si\{^1H\}$ NMR (80 MHz, $[D_8]THF$, 25 °C) of $[(Me_4TACD)RbSiH_3]_2$ (**9**).

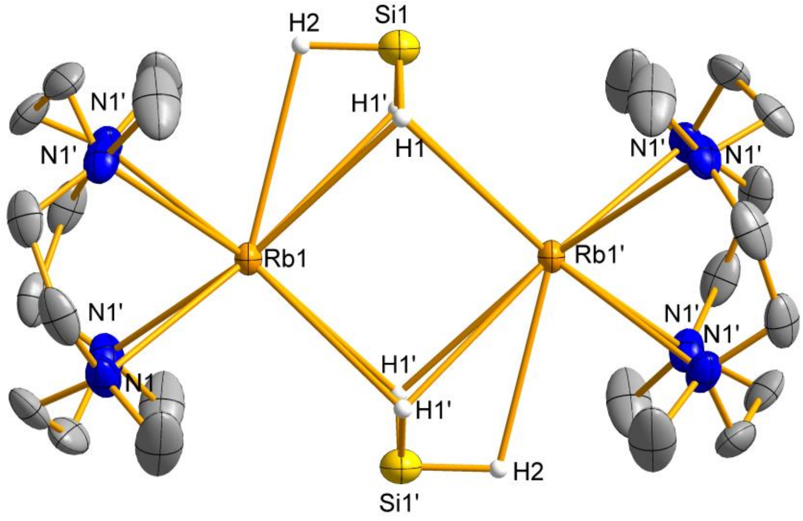

Figure S46. Molecular structures of $[(Me_4TACD)RbSiH_3]_2$ (**9**). Displacement parameters are shown at a 50% probability level. Hydrogen atoms and lattice benzene molecules are omitted for clarity. Selected interatomic distances [Å]: Rb1–Rb1 4.3453(7), Rb1–Si1 3.7085(9), Rb1–H1 3.16(3), Rb1–N1 2.9838(15), Si1–H1 1.39(4), Si1–H2 1.40(6).

2.19. Hydrogenolysis of $[(Me_4TACD)CsSiPh_3]_\infty$ (5)

A degassed solution of $[(Me_4TACD)CsSiPh_3]_\infty$ (31 mg, 0.05 mmol) in THF (2 mL) was charged with H_2 (1 bar) in a glass autoclave and stirred at 25 °C. after 5 d, an off-white precipitate formed. The supernatant was decanted off, the residue washed with pentane (2 x 1 mL) and dried under reduced pressure to give $[CsSiH_3]_\infty$ (5 mg, 0.03 mmol, 63%) as an off-white powder.

^{1}H NMR (400 MHz, [D$_8$]THF, 25 °C): δ = 1.52 (s, $^1J_{Si-H}$ = 74 Hz, SiH_3) ppm.

Insolubility in aliphatic, aromatic hydrocarbons and ethereal solvents prevented from recording ^{29}Si{^{1}H} NMR spectra.

2.20. ^{1}H NMR spectrum of $[CsSiH_3]_\infty$

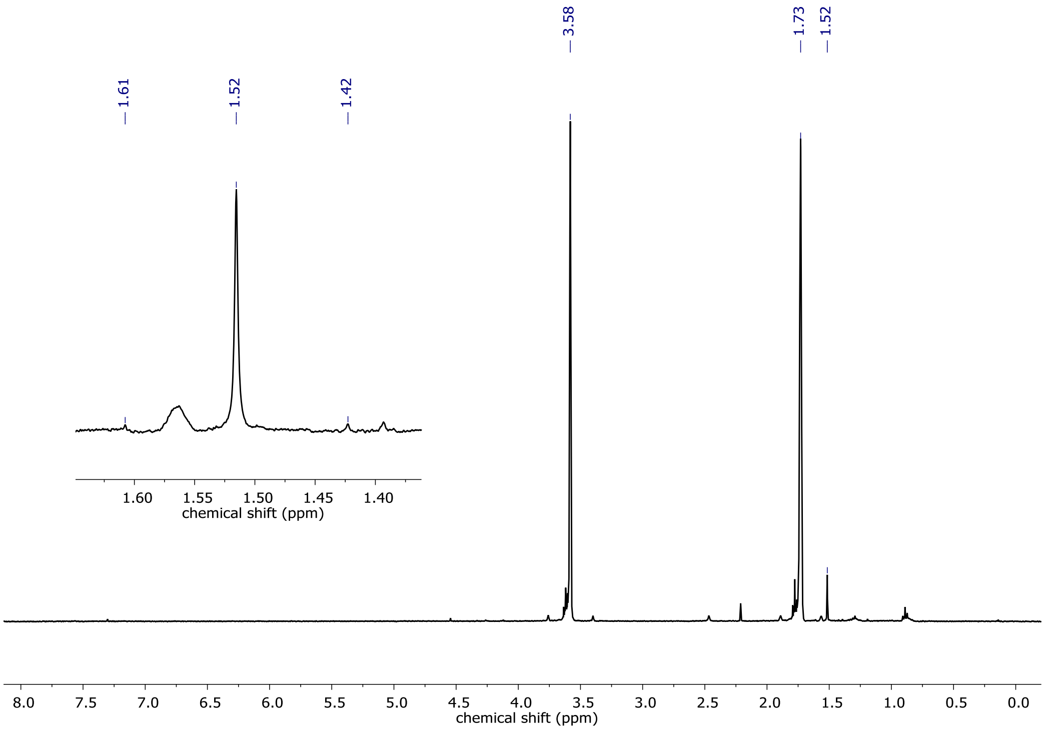

Figure S47. ^{1}H NMR (400 MHz, [D$_8$]THF, 25 °C) of $[CsSiH_3]_\infty$ (**9**).

3. X-Ray Crystallography

X-ray diffraction data were collected at –173 °C in ω-scan mode on an Eulerian 4-circle diffractometer STOE STADIVARI (**1**, **2**, **3**, **4**, **8**) or on a Bruker D8 goniometer with an APEX CCD area-detector (**[THF]-3**, **5**, **7**, **9**). The structures were solved by direct methods using SHELXT 2014/4,[S6] except for the crystal structures of **2** and **9** that could be solved by isotypic replacement using the coordinates of **1** and **8**, respectively. All refinements were carried out against F^2 with SHELXL-2013[S7] as implemented in the program system Olex2.[S8] Refinement results are given in Table S1.

The packing of **9** contains co-crystallized benzene. The molecular dimers in **8** and in **9** show crystallographic site symmetry *m.mm* around Wckoff position 2*a* leading to disorder (split positions) of the CH_2 groups of the Me_4TACD ligands, as well as crystallographic site symmetry *m* around Wyckoff position 8*j* for the co-crystallized benzene molecules. Disorder was also observed and resolved by using split positions for the atoms of the CH_2 units of the Me_4TACD ligand C1-C8 in **1** and in **2**, as well as for all atoms of the Me_4TACD ligand in **3**. For the refinement of **5**, the reflections 0 0 1 and 0 0 -1 were omitted from the refinement, because they were most likely affected by the beam stop. The structure of **1** was refined as a two-component twin using the twin matrix -1 0 0 0 1 0 0 0 -1 with fractional contribution of the twin components of 0.62 and 0.38. Similarity restraints (using the command SIMU as implied in SHELXL) were applied in the refinement for the split positions of the atoms N1-N4 and C1-C8 in **3**. Except for the structure of **7**, all non-hydrogen atoms were refined with anisotropic displacement parameters. The hydrogen atoms were included in calculated positions and treated as riding throughout the refinement. Only the hydride atoms H1 and H2 attached to Si1 in **8** and in **9** were located in a Fourier difference map and refined in their position. Due to imposed crystallographic symmetry, the position of H2 is disordered. Crystallographic data of **7** were only obtained up to a low resolution with θ values up to 20.7°. The molecule shows end-for-end disorder along the crystallographic *c* axis around Wyckoff site position 2*c* with site symmetry 4 in space group *P*4/*n*. Only the nitrogen atoms were refined without split positions, and isotropic parameters were used for all non-hydrogen atoms. Hydrogen atoms were included in calculated positions and treated as riding, except for the three expected SiH_3 hydrogen atoms that were omitted. Because of the low resolution, no crystallographic details such as bond distances and angles are discussed for this compound. Graphical representations were performed with the program DIAMOND.[S9] CCDC-1971855 (**1**), -1971856 (**2**), -1971857 (**3**), -1971858 (**[THF]-3**), -1971859 (**4**), -1971860 (**5**), -1971861 (**7**), -1971862 (**8**) and -1971863 (**9**) contain the supplementary crystallographic data for this paper.

These data can be obtained free of charge from the Crystallographic Data Centre via www.ccdc.cam.ac.uk/data_request/cif.

Table S1. Crystallographic data of **1**, **2** and **3**.

	**1**	**2**	**3**
formula	$C_{30}H_{43}LiN_4Si$	$C_{30}H_{43}N_4NaSi$	$C_{30}H_{43}KN_4Si$
Fw /g·mol^{-1}	494.71	510.76	526.87
cryst. color, habit	yellow block	yellow block	orange plate
crystal size / mm	0.26 × 0.30 × 0.36	0.12 × 0.21 × 0.32	0.15 × 0.20 × 0.25
crystal system	monoclinic	monoclinic	monoclinic
space group	*Cc*	*Cc*	*Cc*
a / Å	11.9450(7)	11.997(2)	14.7235(7)
b / Å	14.8567(7)	15.039(3)	12.5229(5)
c / Å	16.3723(10)	16.753(3)	17.2796(10)
β / °	97.447(5)	97.5508(14)	109.271(4)
V / Å^3	2881.0(3)	2996.5(10)	3007.5(3)
Z	4	4	4
d_{calc}/Mg·m^{-3}	1.141	1.132	1.164
μ(MoKα)/mm^{-1}			0.240
μ(CuKα)/mm^{-1}	0.888	1.005	
F(000)	1072	1104	1136
θ range / °	4.78, 89.40	5.33, 71.77	2.19, 26.37
index ranges	-15≤*h*≤15, -19≤*k*≤19, -21≤*l*≤21	-14≤*h*≤14, -18≤*k*≤17, -15≤*l*≤19	-14≤*h*≤18, -15≤*k*≤15, -21≤*l*≤21
refln.	25406	14079	12251
independ. reflns (R_{int})	6573 (0.0589)	4411 (0.0120)	4426 (0.0637)
observed reflns	4450	4349	3303
data/restr./param	6573 / 8 / 403	4411 / 2 / 402	4426 / 74 / 478
R_1, $wR2$ [$I > 2\sigma(I)$]	0.0481, 0.0897	0.0264, 0.0680	0.0510, 0.0584
R_1, $wR2$ (all data)	0.0804, 0.0966	0.0260, 0.0682	0.0339, 0.0608
GooF on F^2	0.901	1.048	0.837
largest diff. peak, hole/ e·Å^3	0.224, -0.261	0.230, -0.292	0.205, -0.161
CCDC number	1971855	1971856	1971857

Table S1 (continued). Crystallographic data of **[THF]-3**, **4** and **5**.

	**[THF]-3**	**4**	**5**
formula	$C_{34}H_{51}KN_4OSi$	$C_{60}H_{86}N_8Rb_2Si_2$	$C_{30}H_{43}CsN_4Si$
Fw /g·mol^{-1}	598.97	1146.48	620.68
cryst. color, habit	orange block	yellow plate	orange rod
crystal size / mm	0.22 × 0.24 × 0.41	0.04 × 0.14 × 0.21	0.18 × 0.20 × 0.31
crystal system	monoclinic	monoclinic	triclinic
space group	$P2_1/c$	$P2_1/c$	$P1$
a / Å	9.8683(13)	12.219(6)	8.880(3)
b / Å	18.807(3)	15.4252(5)	9.466(3)
c / Å	18.626(2)	16.3691(7)	10.690(3)
α / °			102.932(5)
β / °	93.751(2)	94.475(4)	103.646(5)
γ / °			111.903(5)
V / Å^3	3449.5(8)	3075.9(15)	760.2(4)
Z	4	2	1
d_{calc}/Mg·m^{-3}	1.153	1.238	1.356
μ(MoKα)/mm^{-1}	0.220	1.67	1.281
F(000)	1296	1208	320
θ range / °	1.54, 26.53	1.82, 28.28	2.47, 28.48
index ranges	$-12\leq h\leq 12$, $-23\leq k\leq 23$, $-23\leq l\leq 23$	$-16\leq h\leq 16$, $-20\leq k\leq 19$, $-21\leq l\leq 18$	$-11\leq h\leq 11$, $-12\leq k\leq 12$, $-14\leq l\leq 13$
refln.	41915	28042	8093
independ. reflns (R_{int})	7153 (0.0714)	7601 (0.0661)	6451 (0.0441)
observed reflns	4969	4576	5831
data/restr./param	7153 / 0 / 374	7601 / 0 / 329	6451 / 3 / 329
R_1, $wR2$ [I> 2σ(I)]	0.0707, 0.1753	0.0618, 0.1214	0.0523, 0.0917
R_1, $wR2$ (all data)	0.1005, 0.1936	0.1288, 0.1455	0.0599, 0.0954
GooF on F^2	1.070	1.013	0.989
largest diff. peak, hole/ e·Å^3	0.827, -0.586	1.542, -0.679	0.969, -1.420
CCDC number	1971858	1971859	1971860

Table S1. Crystallographic data of **7**, **8** and **9**.

	**7**	**8**	**9**
formula	$C_{12}H_{28}N_4NaSi$	$C_{24}H_{62}K_2N_8Si_2$, $2(C_6H_6)$	$C_{24}H_{62}N_8Rb_2Si_2$, $2(C_6H_6)$
Fw /g·mol^{-1}	282.49	753.41	846.15
cryst. color, habit	colorless rod	colorless block	yellow block
crystal size / mm	0.13 × 0.13 × 0.24	0.16 × 0.17 × 0.18	0.28 × 0.33 × 0.35
crystal system	tetragonal	tetragonal	tetragonal
space group	$P4/n$	$P4_2/mnm$	$P4_2/mnm$
a / Å	9.77(8)	12.3401(7)	12.3934(11)
b / Å	9.77(8)	12.3401(7)	12.3934(11)
c / Å	9.45(8)	14.9372(11)	14.9632(13)
V / Å^3	901(17)	2274.6(3)	2298.3(5)
Z	2	2	2
d_{calc}/Mg·m^{-3}	1.041	1.10	1.223
μ(MoKα)/mm^{-1}	0.147	0.293	2.214
F(000)	312	824	896
θ range / °	2.16, 20.69	2.14, 30.41	2.13, 30.58
index ranges	$-9 \le h \le 9$, $-9 \le k \le 9$, $-9 \le l \le 9$	$-17 \le h \le 15$, $-13 \le k \le 17$, $-17 \le l \le 19$	$-17 \le h \le 17$, $-16 \le k \le 16$, $-21 \le l \le 20$
refln.	6197	11766	32393
independ. reflns (R_{int})	472 (0.1766)	1761 (0.0528)	1914 (0.0682)
observed reflns	248	1028	1542
data/restr./param	472 / 0 / 38	1761 / 0 / 88	1914 / 0 / 90
R_1, $wR2$ [$I > 2\sigma(I)$]	0.0915, 0.2497	0.0572, 0.1601	0.0320, 0.0764
R_1, $wR2$ (all data)	0.1453, 0.2925	0.1013, 0.1796	0.0458, 0.0811
GooF on F^2	1.137	0.958	1.078
largest diff. peak, hole/ e·Å^3	0.312, -0.327	1.432, -0.318	0.930, -0.352
CCDC number	1971861	1971862	1971863

3.1 Bent $[SiPh_3]^-$ fragments in 1 – 5 and [THF]-3

(1)

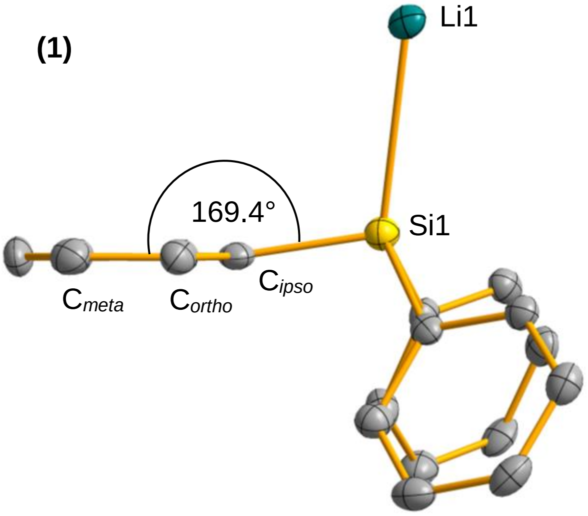

(2)

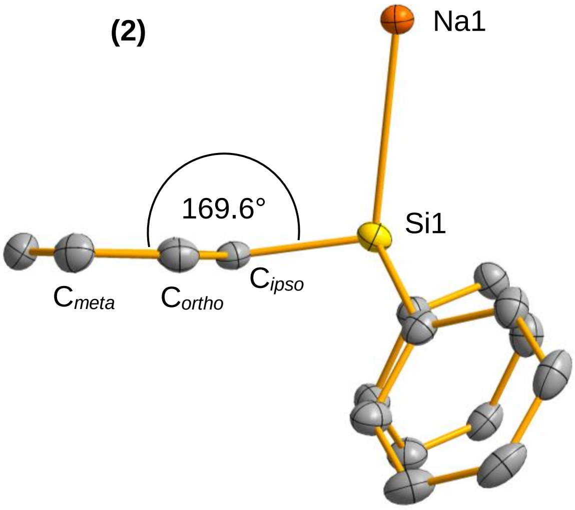

(3)

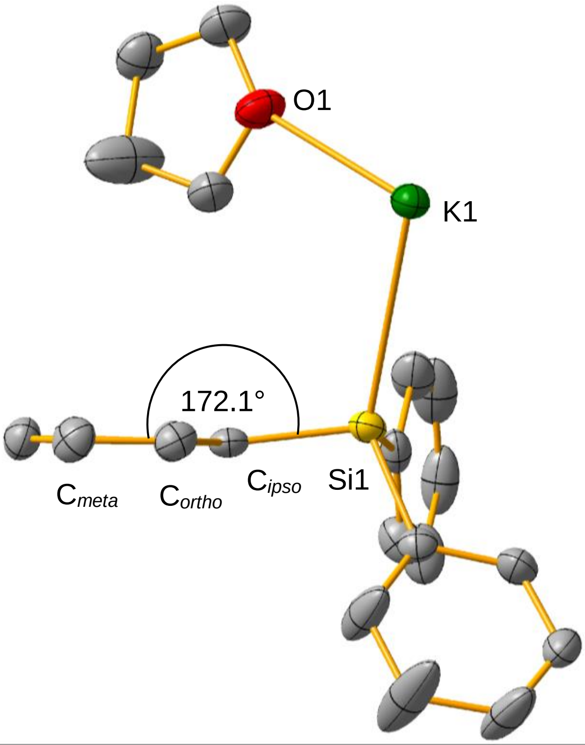

([THF]-3)

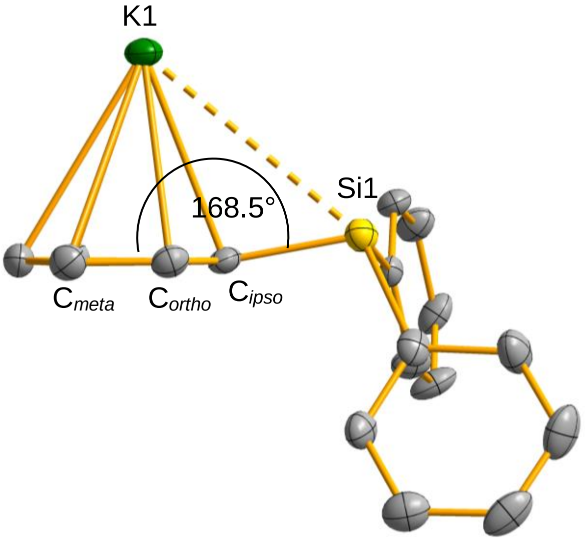

(4)

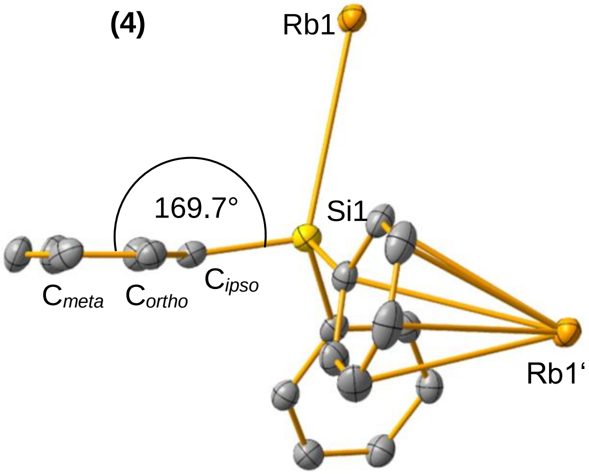
Rb1
169.7°
Si1
C$_{meta}$
C$_{ortho}$
C$_{ipso}$
Rb1‘

(5)

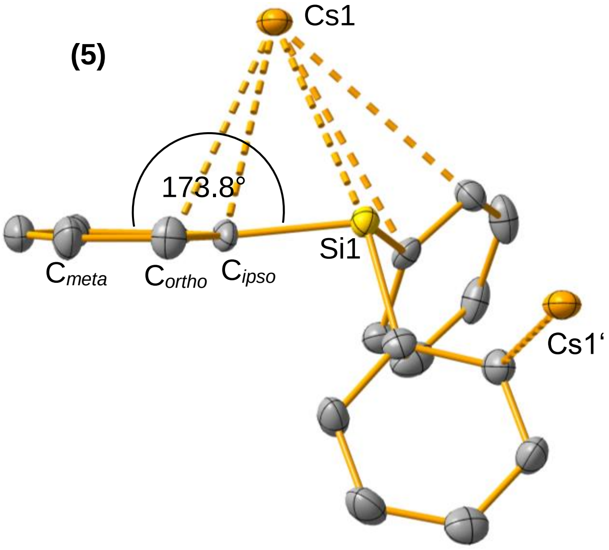
Cs1
173.8°
C$_{meta}$
C$_{ortho}$
C$_{ipso}$
Si1
Cs1‘

4. DFT Calculations

All calculations were carried out at the DFT level of theory using the hybrid functional B3PW91[S10] with the Gaussian 09 suite of programs.[S11] Geometry optimizations were carried out without any symmetry constraints. The enthalpy was determined at T = 298 K in the gas phase and the density was analyzed using the NBO method.[S12] Na, K and Si were represented by a 6-311+G* basis set[S13] and all other atoms (N,C,H) by a 6-31G** one.[S14]

[(Me$_4$TACD)NaSiH$_3$]

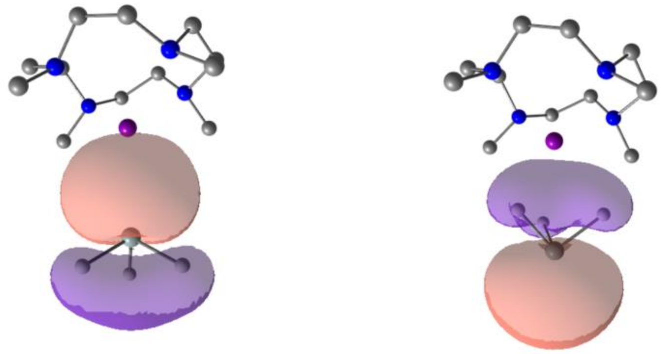

	[(**L**)Na(κSi-SiH$_3$)]	[(**L**)Na(κ^3H-SiH$_3$)]
Distance	Na1-Si1 = 2.91<br>Si-H = 1.54	Na1-Si1 = 2.99<br>Si-H = 1.59
Natural Charges	Na1 = 0.54416<br>Si1 = -0.19740<br>H1 = -0.17787<br>H2 = -0.17641<br>H3 = -0.17628	Na1 = 0.65952<br>Si1 = -0.03117<br>H1 = -0.27211<br>H2 = -0.27337<br>H3 = -0.27355
Natural Electron Configuration	Na1 [core]3S(0.21)4p(0.24)<br>Si1 [core]3S(1.32)3p(2.86)<br>H1 1S(1.18)<br>H2 1S(1.18)<br>H3 1S(1.18)	Na1 [core]3S(0.11)4p(0.22)<br>Si1 [core]3S(1.55)3p(2.46)<br>H1 1S(1.27)<br>H2 1S(1.27)<br>H3 1S(1.27)
Wiberg Bond Index	Na1-Si1 = 0.4547<br>Si1-H1 = 0.9588<br>Na1-H1 = 0.0034	Na1-Si1 = 0.1151<br>Si1-H1 = 0.8574<br>Na1-H1 = 0.0531
Natural Bond Orbital	BD(1) Na1-Si1<br>(13.09%) Na1 s(55.05%)p(44.95%)<br>(86.91%) Si1 s(44.88%)p(55.12%)	Donation of Si-H to Na<br>42,8 Kcal/mol

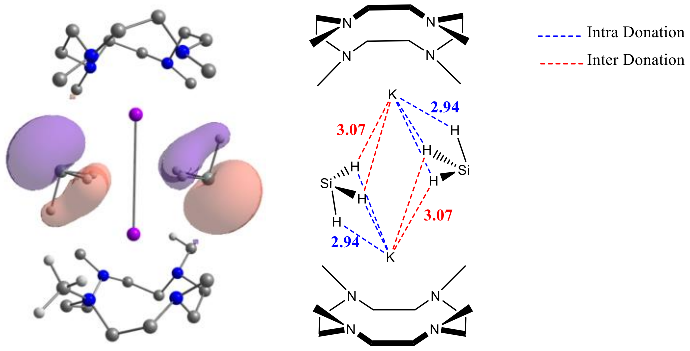

[(**L**)KSiH3]2

Distance	Si1-K1= 3.617 K1···K1' = 4,50<br>Si1-H1 = 1.58 Si1···Si1' = 5,51
Natural Charges	K1 = 0.6799<br>Si1 = -0.12635<br>H1 = -0.24005<br>H2 = -0.23902<br>H3 = -0.22080
Natural Electron Configuration	K1 [core]4S(0.09)3d(0.01)4p(0.23)<br>Si1 [core]3S(1.47)3p(2.64)<br>H1 1S(1.24)<br>H2 1S(1.24)<br>H3 1S(1.22)
Wiberg Bond Index	K1-Si1 = 0.0555<br>Si1-H1 =0.8889<br>K1-H = 0.0282
Natural Bond Orbital	Donation intra Si-H to K = 21,76 kcal/mol<br>Donation inter Si-H to K = 16,08 kcal/mol

[(**L**)Na(κSi-SiH_3)]
49
scf done: -698.787094

Na	7.326129	7.350620	3.719738
Si	7.295350	7.343955	6.624887
N	7.759686	5.187363	2.389830
N	5.239787	6.900745	2.289848
N	9.473646	7.707644	2.345537
N	6.954705	9.418949	2.246263
C	8.275551	4.178359	3.312777
C	6.476755	4.764723	1.823753
C	5.622058	5.912017	1.281301
C	4.185450	6.392975	3.165515
C	8.736549	5.516835	1.352323
C	9.889739	6.397482	1.838757
C	10.496087	8.267696	3.227130
C	9.132797	8.631898	1.264334
C	8.258357	9.809114	1.703111
C	6.405215	10.482262	3.085204
C	6.016437	9.027005	1.194324
C	4.844109	8.176199	1.688055
H	10.159790	9.217545	3.649018
H	10.668153	7.592193	4.069174
H	11.455348	8.441045	2.706372
H	3.980434	7.119119	3.956530
H	3.246915	6.183354	2.620825
H	4.507854	5.472815	3.658113
H	5.464196	10.163604	3.539594
H	7.094752	10.699129	3.905379
H	6.218877	11.413870	2.520645
H	9.201775	4.523241	3.777808
H	7.558917	4.017077	4.122170
H	8.477107	3.212430	2.814835
H	9.171832	4.601633	0.906581
H	8.213340	6.025428	0.536082
H	10.414999	5.884214	2.650204
H	10.623161	6.490807	1.015519
H	8.781711	10.373812	2.480905
H	8.154746	10.500215	0.845384
H	10.042815	9.043291	0.786492
H	8.614149	8.069220	0.481461
H	4.287117	8.736987	2.445114
H	4.144192	8.031615	0.843381
H	5.600368	9.913754	0.678472
H	6.569440	8.470622	0.430607
H	6.171665	6.426903	0.486621
H	4.730404	5.472180	0.794331
H	6.610232	4.022604	1.013851
H	5.924166	4.247692	2.614464
H	7.535181	6.009151	7.350061
H	8.315863	8.194566	7.397728
H	6.031794	7.777741	7.384396

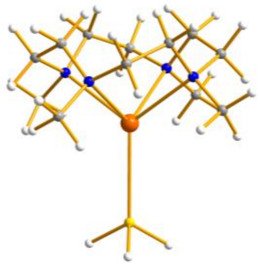

[(**L**)Na(κ^3H-SiH_3)]
49
scf done: -698.782980

Na	7.331708	7.323058	3.752617
Si	7.206815	7.696759	6.724696
N	7.762148	5.159582	2.401920
N	5.245560	6.873627	2.335754
N	9.482625	7.673930	2.396528
N	6.961637	9.393975	2.331179
C	8.282843	4.125064	3.293908
C	6.477767	4.747073	1.829068
C	5.622767	5.903851	1.307601
C	4.186592	6.354132	3.199938
C	8.734524	5.507437	1.364619
C	9.893715	6.374095	1.860374
C	10.513437	8.219580	3.278300
C	9.135922	8.618518	1.333352
C	8.266680	9.789721	1.796492
C	6.416507	10.444047	3.190725
C	6.020972	9.021062	1.275098
C	4.850595	8.162447	1.760306
H	10.169455	9.147325	3.740405
H	10.710989	7.518089	4.092838
H	11.460274	8.422283	2.745596
H	4.004399	7.054481	4.019174
H	3.242155	6.181590	2.652713
H	4.493887	5.409376	3.654480
H	5.472924	10.121272	3.636073
H	7.104629	10.634214	4.018213
H	6.239249	11.387120	2.642686
H	9.212926	4.456060	3.761296
H	7.571058	3.941668	4.102834
H	8.479263	3.173158	2.767886
H	9.162243	4.599370	0.897850
H	8.207604	6.034268	0.562674
H	10.424433	5.843304	2.656936
H	10.620580	6.482674	1.033267
H	8.791368	10.335144	2.586822
H	8.166453	10.498733	0.953029
H	10.045008	9.035193	0.858667
H	8.612156	8.070620	0.543347
H	4.301468	8.709448	2.532753
H	4.144063	8.034258	0.918592
H	5.602157	9.916310	0.776580
H	6.570814	8.478307	0.499133
H	6.170388	6.432237	0.520442
H	4.729795	5.472909	0.815407
H	6.612035	4.018820	1.006995
H	5.925549	4.216629	2.611179
H	7.700908	6.384985	5.972671
H	8.067148	8.608262	5.746648
H	5.951506	7.807628	5.755146

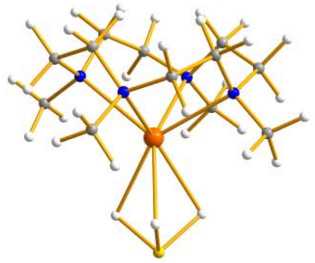

$[(L)KSiH_3]_2$

98

scf done: -1453.849892

K	7.732895	4.558461	7.370510
K	4.551410	7.734961	7.583889
Si	5.965668	6.287464	4.729797
Si	6.284513	5.971807	10.224759
N	8.001576	1.906561	6.047278
N	8.149680	2.214725	9.108011
N	10.336662	4.452124	8.796844
N	2.205681	8.146487	5.848166
N	4.439352	10.337705	6.155881
N	4.136880	10.166076	9.214703
N	1.899740	7.999680	8.909176
N	10.164054	4.147027	5.738636
C	10.327950	5.404275	5.014092
C	9.859742	3.069550	4.794630
C	9.318072	1.783144	5.421705
C	7.765448	0.850783	7.033772
C	8.526735	1.025210	8.350375
C	9.172139	2.613678	10.080522
C	10.467270	3.165005	9.481298
C	11.427711	4.676422	7.848156
C	11.336850	3.855300	6.559657
C	6.877033	2.015906	9.798514
C	10.267897	5.545172	9.764794
C	6.945710	1.910208	5.038605
C	2.601933	9.169009	4.874639
C	3.151356	10.465565	5.472569
C	4.662578	11.429467	7.103984
C	3.842655	11.337931	8.393207
C	3.060558	9.860645	10.159676
C	1.774621	9.316438	9.533827
C	0.843728	7.761119	7.923523
C	1.015949	8.521845	6.606317
C	2.008753	6.872973	5.158698
C	5.394379	10.332461	9.938245
C	1.905708	6.944567	9.918610
C	5.531607	10.270567	5.186937
H	6.928897	6.934828	9.150830
H	5.315530	5.356571	9.139295
H	5.244219	7.033083	10.680892
H	6.928767	6.933816	5.802487
H	5.352660	5.318340	5.816444
H	7.027935	5.248264	4.273450
H	6.587328	2.942157	10.305620
H	6.087612	1.765474	9.083799
H	6.928353	1.200083	10.544309
H	7.078215	2.745558	4.346612
H	6.914116	0.971364	4.453809
H	5.972570	2.048285	5.519250
H	10.528292	6.223848	5.709670
H	11.161002	5.366972	4.286827
H	9.403920	5.646409	4.481447
H	9.400371	5.425533	10.420162
H	11.179415	5.613163	10.388394
H	10.139557	6.496915	9.240272
H	2.741290	7.078919	10.609963
H	0.967285	6.911911	10.504026
H	2.045042	5.971302	9.438587
H	5.638512	9.409081	10.471107
H	6.213070	10.533946	9.241958
H	5.356178	11.165765	10.665174
H	2.935069	6.584818	4.650841
H	1.760647	6.083515	5.874179
H	1.192089	6.922178	4.413684
H	12.273226	4.017281	5.989594
H	11.326541	2.792057	6.815074
H	11.428715	5.742072	7.595252
H	12.416098	4.477249	8.307908
H	11.208048	3.235783	10.301746
H	10.881595	2.438539	8.776048
H	8.718305	3.374444	10.725128
H	9.445578	1.767005	10.741775
H	8.378186	0.105282	8.949998
H	9.598637	1.069552	8.137993
H	6.690412	0.830912	7.241683
H	8.011207	-0.149455	6.625779
H	9.303291	1.008948	4.629442
H	10.025714	1.418644	6.172149
H	9.122774	3.461382	4.085515
H	10.750588	2.798866	4.193015
H	5.412907	9.402588	4.532003
H	5.597352	11.181967	4.562919
H	6.484081	10.144150	5.710593
H	1.408574	10.022936	8.783065
H	1.000939	9.301046	10.326582
H	3.454074	9.124830	10.869052
H	2.788823	10.751474	10.760842
H	4.003665	12.274882	8.962601
H	2.779212	11.325855	8.138704
H	5.728432	11.432310	7.356031
H	4.461448	12.417359	6.644004
H	3.220013	11.205948	4.651585
H	2.424760	10.879013	6.178205
H	3.363077	8.716186	4.229760
H	1.754237	9.440420	4.213862
H	0.095924	8.371204	6.007367
H	1.058472	9.593967	6.817945
H	0.825548	6.685914	7.716329
H	-0.156666	8.005429	8.332018

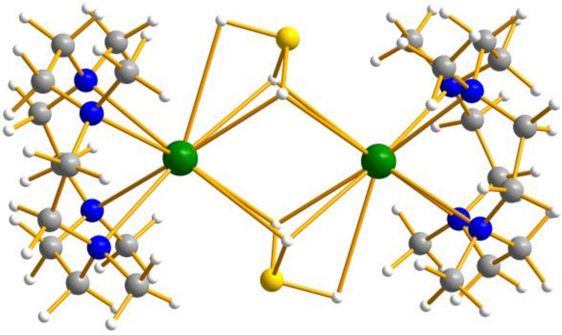

5. References

[S1] a) V. Leich, T. P. Spaniol, L. Maron, J. Okuda, *Angew. Chem. Int. Ed.* **2016**, *55*, 4794-4797. b) A. Causero, G. Ballmann, J. Pahl, H. Zijlstra, C. Farber, S. Harder, *Organometallics* **2016**, *35*, 3350-3360. c) A. S. S. Wilson, M. S. Hill, M. F. Mahon, *Organometallics* **2018**, *38*, 351-360. d) M. S. Hill, M. F. Mahon, A. S. S. Wilson, C. Dinoi, L. Maron, E. Richards, *Chem. Commun.* **2019**, *55*, 5732-5735.

[S2] J. H. Coates, D. A. Hadi, S. F. Lincoln, *Aust. J. Chem.* **1982**, *35*, 903.

[S3] G. Maier, H. P. Reisenauer, A. Meudt, *Eur. J. Org. Chem.* **1998**, *1998*, 1285-1290.

[S4] J. W. Connolly, G. Urry, *Inorg. Chem.* **1963**, *2*, 645-646.

[S5] M. H. Chisholm, S. R. Drake, A. A. Naiini, W. E. Streib, *Polyhedron* **1991**, *10*, 337-345.

[S6] a) A. Altomare, G. Cascarano, C. Giacovazzo, A. Guagliardi, *J. Appl. Crystallogr.* **1993**, *26*, 343-350. b) G. M. Sheldrick, *Acta Cryst. A* **2015**, *71*, 3-8.

[S7] G. M. Sheldrick, *Acta Cryst. A* **2008**, *64*, 112-122.

[S8] O. V. Dolomanov, L. J. Bourhis, R. J. Gildea, J. A. K. Howard, H. Puschmann, *J. Appl. Crystallogr.* **2009**, *42*, 339-341.

[S9] H. Putz, K. Brandenburg, *Diamond - Crystal and Molecular Structure Visualization, Crystal Impact,* Bonn, **2017**.

[S10] a) A. D. Becke, *J. Chem. Phys.* **1993**, *98*, 5648-5652. b) J. P. Perdew, Y. Wang, *Phys. Rev. B* **1992**, *45*, 13244-13249.

[S11] M. J. Frisch, G. W. Trucks, H. B. Schlegel, G. E. Scuseria, M. A. Robb, J. R. Cheeseman, G. Scalmani, V. Barone, B. Mennucci, G. A. Petersson, H. Nakatsuji, M. Caricato, X. Li, H. P. Hratchian, A. F. Izmaylov, J. Bloino, G. Zheng, J. L. Sonnenberg, M. Hada, M. Ehara, K. Toyota, R. Fukuda, J. Hasegawa, M. Ishida, T. Nakajima, Y. Honda, O. Kitao, H. Nakai, T. Vreven, J. A. Montgomery, Jr., J. E. Peralta, F. Ogliaro, M. Bearpark, J. J. Heyd, E. Brothers, K. N. Kudin, V. N. Staroverov, R. Kobayashi, J. Normand, K. Raghavachari, A. Rendell, J. C. Burant, S. S. Iyengar, J. Tomasi, M. Cossi, N. Rega, J. M. Millam, M. Klene, J. E. Knox, J. B. Cross, V. Bakken, C. Adamo, J. Jaramillo, R. Gomperts, R. E. Stratmann, O. Yazyev, A. J. Austin, R. Cammi, C. Pomelli, J. W. Ochterski, R. L. Martin, K. Morokuma, V. G. Zakrzewski, G. A. Voth, P. Salvador, J. J. Dannenberg, S. Dapprich, A. D. Daniels, O. Farkas, J. B. Foresman, J. V. Ortiz, J. Cioslowski, D. J. Fox, Gaussian 09; D.02 ed. Pittsburgh PA 2009.

[S12] A. E. Reed, L. A. Curtiss, F. Weinhold, *Chem. Rev.* **1988**, *88*, 899-926.

[S13] A. D. McLean, G. S. Chandler, *J. Chem. Phys.* **1980**, *72*, 5639-5648.

[S14] W. J. Hehre, R. Ditchfield, J. A. Pople, *J. Chem. Phys.* **1972**, *56*, 2257-2261.
